# Ionic Liquid Coating‐Driven Nanoparticle Delivery to the Brain: Applications for NeuroHIV

**DOI:** 10.1002/advs.202305484

**Published:** 2024-04-04

**Authors:** Christine M. Hamadani, Fakhri Mahdi, Anya Merrell, Jack Flanders, Ruofan Cao, Priyavrat Vashisth, Gaya S. Dasanayake, Donovan S. Darlington, Gagandeep Singh, Mercedes C. Pride, Wake G. Monroe, George R. Taylor, Alysha N. Hunter, Gregg Roman, Jason J. Paris, Eden E. L. Tanner

**Affiliations:** ^1^ Department of Chemistry & Biochemistry The University of Mississippi University MS 38677 USA; ^2^ Department of BioMolecular Sciences The University of Mississippi University MS 38677 USA

**Keywords:** brain delivery, cellular hitchhiking, ionic liquids, nanoparticles, red blood cells

## Abstract

Delivering cargo to the central nervous system (CNS) remains a pharmacological challenge. For infectious diseases such as HIV, the CNS acts as a latent reservoir that is inadequately managed by systemic antiretrovirals (ARTs). ARTs thus cannot eradicate HIV, and given CNS infection, patients experience neurological deficits collectively referred to as “neuroHIV”. Herein, the development of bioinspired ionic liquid‐coated nanoparticles (IL‐NPs) for in situ hitchhiking on red blood cells (RBCs) is reported, which enables 48% brain delivery of intracarotid arterial‐ infused cargo. Moreover, IL choline trans‐2‐hexenoate (CA2HA 1:2) demonstrates preferential accumulation in parenchymal microglia over endothelial cells post‐delivery. This study further demonstrates successful loading of abacavir (ABC), an ART that is challenging to encapsulate, into IL‐NPs, and verifies retention of antiviral efficacy in vitro. IL‐NPs are not cytotoxic to primary human peripheral blood mononuclear cells (PBMCs) and the CA2HA 1:2 coating itself confers notable anti‐viremic capacity. In addition, in vitro cell culture assays show markedly increased uptake of IL‐NPs into neural cells compared to bare PLGA nanoparticles. This work debuts bioinspired ionic liquids as promising nanoparticle coatings to assist CNS biodistribution and has the potential to revolutionize the delivery of cargos (i.e., drugs, viral vectors) through compartmental barriers such as the blood‐brain‐barrier (BBB).

## Graphical abstract

Ionic liquid‐coated PLGA nanoparticles (IL‐NPs) drive selective affinity to red blood cells after intravascular injection, thereby mediating highly efficient delivery through the blood brain barrier (BBB) via vascular capillary shear. Once entered into the brain, IL‐NPs overcome passive diffusion and are actively transported to resident microglia for internalization to access neuro‐HIV reservoirs.

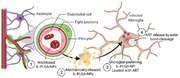

## 1 Introduction

### 1.1 Drug Delivery Barriers Limiting Antiretroviral Therapy (ART) to the Brain may be Overcome via Red Blood Cell (RBC) Hitchhiking In Situ

HIV is present in the CNS soon after infection and establishes a reservoir of latently‐infected microglia, the long‐lived immune cells that reside therein.^[^ ^1^, ^2^, ^3^, ^4^, ^5^ ^]^ Astrocytes are also infected, albeit in a much lesser proportion and their capacity for replication‐competent activation is controversial.^[^ ^6^, ^7^, ^8^, ^9^ ^]^ This reservoir, and others throughout the body, are not effectively treated by ART.^[^ ^10^, ^11^ ^]^ In the CNS, ART is impeded by blood‐brain barrier (BBB) efflux,^[^ ^12^, ^13^ ^]^ and preferentially accumulates in off‐target cell types (e.g., endothelial cells),^[^ ^14^ ^]^ reducing the capacity for therapeutic concentrations to be achieved in cells comprising the central reservoir, such as microglia.^[^ ^15^, ^16^ ^]^ Overcoming viral latency has posed an additional challenge that has found some recourse in novel strategies of latency reversing agents (LRAs) and genome‐editing techniques that may eradicate HIV.^[^ ^17^, ^18^, ^19^, ^20^ ^]^ However, even if successful, these compounds and constructs need to be targeted to HIV reservoirs, which remains to be achieved.

To overcome these challenges, we developed an ionic liquid‐coated nanoparticle (IL‐NP) as a delivery vehicle to transport ART to the brain. Ionic liquids (ILs) are composed of bulky, asymmetric anions and cations, and are liquid at temperatures <100 °C.^[^ ^21^ ^]^ When they are synthesized from bioinspired components, they retain high biocompatibility and have been used in a variety of drug delivery applications, including transdermal, buccal, subcutaneous, and oral delivery of therapeutics.^[^ ^22^, ^23^, ^24^, ^25^ ^]^ We have previously shown that choline trans‐2‐hexenoate (CA2HA 1:2) can be used to coat polymeric nanoparticles, and when mixed with whole blood, show spontaneous attachment (“hitchhiking”) onto red blood cells (RBCs). When intravenously (IV) injected into the tail vein, 46.6 ± 13.5% (n = 6, ± standard deviation) of the delivered dose accumulated in the lungs, the first encountered capillary bed from systemic circulation.^[^ ^26^, ^27^ ^]^ Based on past work by Muzykantov and other colleagues,^[^ ^28^, ^29^, ^30^, ^31^ ^]^ we hypothesized that injection into the carotid artery would instead confer similar rates of brain accumulation, due to RBC shearing behavior through the BBB.

## 2 Results and Discussion

### 2.1 Nanoformulation and Characterization of IL‐PLGA NPs for Brain Delivery of ART

We first prepared carboxylic acid‐terminated poly(lactic‐co‐glycolic) acid (PLGA)‐based nanoparticles (NPs) by nanoprecipitation and solvent evaporation of acetonitrile (ACN) (Figure S1, Supporting Information), as previously described and as detailed in the Experimental Section.^[^ ^26^ ^]^ This produced bare, unloaded particles of 45.1 ± 4.8 nm hydrodynamic diameter, −26.6 ± 5.2 mV surface charge, and 0.17 ± 0.06 polydispersity index (PDI) by Dynamic Light Scattering (DLS, n = 4). We then loaded our particles with either equivalent amounts of abacavir (ABC) or far‐red fluorescent dye 1,1′‐dioctadecyl‐3,3,3′,3′‐ tetramethylindodicarbocyanine, 4‐chlorobenzenesulfonate (DiD) at ≈2% by weight of the polymer (normalized to cargo molecular weight) into the organic phase. ABC‐loaded bare PLGA NPs (n = 7) had diameters of 76.4 ± 13.5 nm, a stable surface charge of −36.9 ± 9.8 mV, and a monodisperse PDI of 0.09 ± 0.04, while the DiD‐loaded bare PLGA NPs (n = 3) were 61.6 ± 1.3 nm, −24.8 ± 0.26 mV, and 0.11 ± 0.015 PDI, indicating slightly improved loading abilities for ABC over DiD.

**Figure 1:**
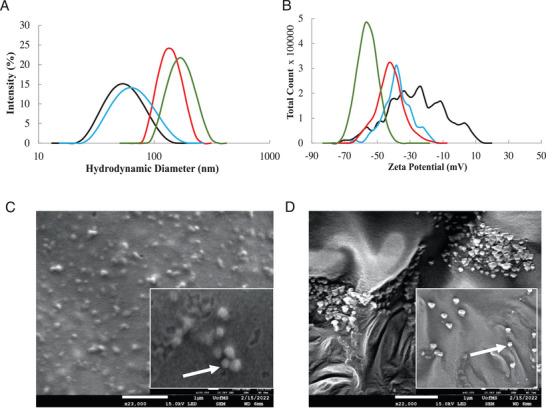
Ionic liquid coats both empty PLGA NPs and those loaded with 60 µg mL^−1^ abacavir. Empty bare PLGA (black line) and IL‐coated PLGA (red line) NPs undergo an increase in size A) and anionic shift in surface charge B) when abacavir is loaded into Bare PLGA (blue line) and IL‐coated PLGA (green line) NPs. Scanning Electron Microscopy (SEM) of Bare PLGA and C) IL‐coated PLGA D) shows morphological changes upon IL coating. Scale = 1 µm.

The loaded bare PLGA NPs were then coated with choline 2‐hexenoate (CA2HA 1:2) IL (IL‐PLGA NPs) by placing a single ≈10 mg liquid drop in the center of the vial (10 mg neat IL/mg PLGA) and were stirred for two more hours. In each case, the previously bare PLGA NPs increased in size, and decreased in surface charge while maintaining a monodisperse PDI below 0.2. ABC‐loaded IL‐coated NPs were 191.5 ± 23.6 nm, −54.8 ± 6.5 mV, and had a PDI of 0.12 ± 0.07 (n = 5). **Figure** **1** shows the size (A) and surface charge (B) of the bare empty PLGA (black), ABC‐loaded PLGA (blue), IL‐coated empty PLGA (red), and ABC‐loaded IL‐coated PLGA (green), as well as C) bare and D) IL‐coated NP morphology by Scanning Electron Microscopy (SEM). Importantly, the roles of the 2HA anion and choline cation in the IL coating were independently validated, in triplicate, for both self‐assembly and RBC hitchhiking on PLGA DiD NPs. Briefly, PLGA DiD NPs were coated with either: 1) 5.8 mg of purified 2HA anion and 4.2 mg of choline bicarbonate (to form CA2HA 1:2 in situ), 2) 5.8 mg of purified 2HA anion, and 3) 4.2 mg of choline bicarbonate. These three variant formulations of CA2HA 1:2 PLGA DiD NPs were evaluated by DLS (n = 3) (Table S1, Supporting Information) and also comparatively tested for RBC hitchhiking in whole human blood (n = 3) (Figure S2, Supporting Information), revealing that coating with neat IL is imperative (as opposed to just cation, just anion, or simultaneously adding both). Notably, when self‐assembled together with 2HA, without the influence of the bicarbonate counterion, choline plays a stabilizing role in the coating, while 2HA drives specific outer‐layer interfacial interactions with the biological environment, namely affinity to RBCs in whole blood. Full DLS data is detailed in Table S1 (Supporting Information).

The encapsulation efficiency (EE) and 32‐hour release profile of DiD was determined by fluorescent plate reader (Figure S3, Supporting Information) (DiD, 2% (v/v): 60.43 ± 2.03% (n = 3)), while the presence of IL coating and ABC encapsulation was measured by quantitative ^1^H NMR spectroscopy (Figure S4, Supporting Information) (ABC, 2% (v/v), estimated ≈62.7 µg mL^−1^ from an added 74.1 µg mL^−1^, or ≈84.6% EE). The quantitative difference in encapsulation efficiency discovered between DiD and ABC is consistent with the DLS findings (Figure 1).

### 2.2 ART‐Encapsulated IL‐PLGA NPs Suppress HIV Viral Replication, Enhance Cellular Uptake of, and are Biocompatible to, Human Peripheral Blood Mononuclear Cells (PBMCs)

**Figure 2:**
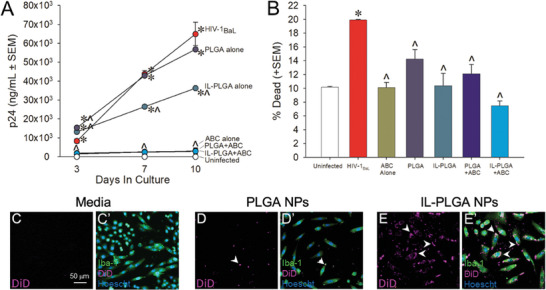
IL‐coated PLGA NPs encapsulate abacavir (ABC), suppress viral replication in HIV‐1 treated human PBMCs without cytotoxicity, and show enhanced human microglia uptake in vitro. A) HIV‐1_BaL_ viral replication (n = 2) is attenuated by CA2HA 1:2‐coated PLGA NPs loaded with abacavir (ABC; 60 µg mL^−1^; n = 3), free ABC alone (60 µg mL^−1^) (n = 3), or CA2HA 1:2‐coated empty PLGA NPs (n = 3). ^*^ indicates significant difference from mock‐infected cells (n = 2); ^ indicates significant difference from HIV‐infected cells; *p* < 0.05 (Repeated‐Measures ANOVA). B) Bare and IL‐coated PLGA NPs with ABC (60 µg mL^−1^) show little cytotoxicity when compared to mock‐infected PBMCs. ^*^ indicates significant difference from mock‐infected cells; ^ indicates significant difference from HIV‐1 infected cells; *p* < 0.05 (One‐Way ANOVA). C–E’) Immunocytochemistry on cultured primary human microglia : C–C’) Media‐control, D–D’) Bare PLGA NPs loaded with DiD (purple), and E–E’) IL‐PLGA NPs loaded with DiD. Cells were co‐labeled with anti‐Iba‐1 (green) and Hoechst nuclear stain (blue). Intracellular DiD (purple) accumulation was qualitatively greater when PLGA‐NPs were coated with IL (see E‐E’). Scale = 50 µm.

To assess the bioactivity of ART when encapsulated inside bare PLGA & IL‐PLGA NPs, HIV‐1 replication was assessed in human peripheral blood mononuclear cells (PBMCs) that were mock‐infected or were infected with HIV‐1_BaL_ (1 ng mL^−1^) for 10 days. PBMCs were treated with 1 mg mL^−1^ bare or IL‐coated PLGA NPs that were either unloaded or loaded with abacavir (ABC, 60 µg mL^−1^). Free ABC drug was also administered alone as a control. Concentration of the HIV‐1 capsid protein (p24, ng mL^−1^) was assessed on days 3, 7, and 10 post‐infection by enzyme‐linked immunosorbent assay (ELISA) (**Figure** **2**A). As expected, viral replication was significantly greater in cells treated with HIV‐1 alone, empty NPs, or empty IL‐coated NPs compared to those that were mock‐infected (*p* < 0.0001–0.0009). Compared to HIV‐infected cells, ABC significantly attenuated viral replication when administered alone and retained its bioactivity when encapsulated in NPs (*p* < 0.0001). Intriguingly, IL‐coated NPs without cargo significantly attenuated HIV‐1 replication on their own (*p* = 0.04); while ILs have previously demonstrated to exert virucidal effects,^[^ ^32^, ^33^, ^34^, ^35^, ^36^ ^]^ this has not been previously demonstrated with HIV‐1.

PBMC viability was also assessed at the 10‐day timepoint via a trypan blue exclusion assay (Figure 2B). As expected, HIV‐1 significantly increased the proportion of dead cells (*p* = 0.0004);^[^ ^37^ ^]^ any other treatment significantly attenuated this (*p* = 0.0002–0.02). Additionally, when visualized by fluorescent microscopy, the uptake of DiD far‐red fluorescent dye in co‐cultured primary human astrocytes and primary human microglial cells (Figure S5, Supporting Information), or primary human microglia alone (Figure 2C–E’), was dramatically enhanced when carried by IL‐PLGA NPs (Figure 2E,E’) compared to bare PLGA NPs (Figure 2D,D’) or media alone (Figure 2C,C’). This increased uptake could be partially explained by lipid extraction behavior engaged by the choline 2‐hexenoate coating, which has been previously found to increase PLGA NP accumulation in RAW 264.7 macrophages while bypassing filopodia‐mediated uptake.^[^ ^27^ ^]^ Alternatively, the carboxylic trans‐2‐hexenoate anion may facilitate uptake via selective interaction with the monocarboxylate transporter (MCT) family, present as MCT‐1 on both RBCs (Figure S6, Supporting Information), as well as on astrocytic and glial cell surfaces.^[^ ^38^, ^39^, ^40^ ^]^

### 2.3 Carotid Arterial Injection Directs IL‐PLGA NPs to the Brain and Results in Regional Abacavir (ABC) Brain Accumulation

To test in vivo delivery efficacy, DiD‐loaded bare and IL‐coated PLGA NPs were injected into the carotid artery (500 µL) of healthy, 8‐week‐old, female, Sprague Dawley rats with in‐dwelling carotid catheters (n = 4/group). 6 hours post‐injection, the rats were sacrificed: whole blood was collected by cardiac puncture followed by transcardial perfusion with phosphate buffered saline (1x PBS pH 7.4). Blood components were immediately analyzed by fluorescence activated cell sorting (FACS) (Figure S7, Supporting Information). Blood‐filtering organs were subsequently harvested (brain, lung, heart, liver, spleen, and kidneys) (n = 3/group). From each treatment group, one animal underwent transcardial perfusion with 1x PBS pH 7.4, additionally followed by 4% (w/v) paraformaldehyde (PFA). One fixed brain from each group was used for epifluorescent imaging while the other three sets of organs were flash frozen and stored at −80 °C to perform biodistribution analysis. No physiologically‐adverse effects of NPs were observed during live study or post‐mortem.

**Figure 3:**
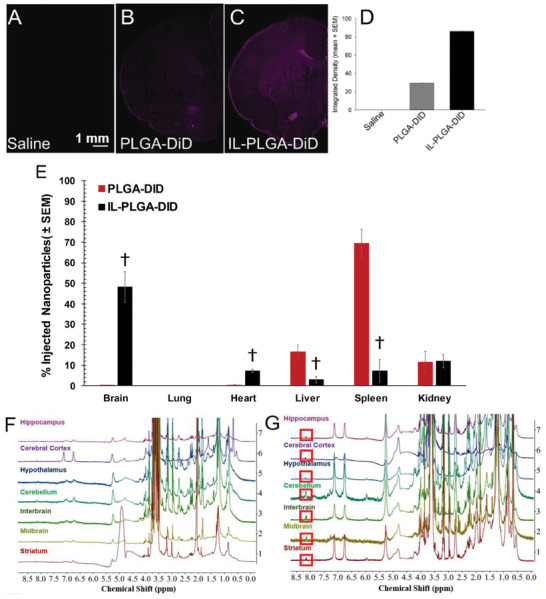
IL‐NPs dramatically enhance delivery to the brain in vivo and influence regional abacavir accumulation. A–D) Sprague‐Dawley rat brain cross‐sections shown after treatment with: (A) Saline, (B) Bare PLGA NPs loaded with DiD (purple), or (C) IL‐coated PLGA NPs loaded with DiD. Scale bar = 1 mm. (D) Signal quantified by densitometry (area × mean intensity; n = 1/group). E) Biodistribution (%) of injected DiD in isolated organs (% ID organ, n = 3/group; mean ± SEM). † denotes significant difference from respective PLGA‐DiD‐treated group; *p* < 0.05 (paired two‐tailed *t*‐test for means). F,G) Representative differences, by ^1^H‐NMR spectroscopy, in abacavir (ABC) regional brain accumulation in Sprague‐Dawley rat brains (n = 3/group) post intra‐carotid injection for (F) empty IL‐PLGA NPs and (G) IL‐PLGA NPs loaded with ABC. Key proton peak for ABC presence at 8.1 ppm is indicated (see red box, panel G).

As shown in **Figure** **3**, notable differences in raw DiD signal were observed for IL‐coated versus uncoated DiD PLGA NPs in the brain, via wide‐field epifluorescence images (Figure 3A–C). Compared to saline‐infused rats (Figure 3A), a faint DiD signal was detected in those infused with DiD‐loaded bare PLGA NPs (Figure 3B). In contrast, a much more intense signal was observed for rats infused with IL‐coated PLGA DiD NPs (Figure 3C; densitometric quantification in Figure 3D). Figure 3E shows the results of the quantitative biodistribution study (n = 3/group, ± SEM). Bare PLGA NPs accumulated primarily in the spleen (69.6 ± 6.9%), with a smaller amount in the liver (16.6 ± 3.3%), kidneys (11.6 ± 5.2%), and the least in the brain (0.1 ± 0.1%). In contrast, IL‐coated PLGA NPs demonstrated the greatest accumulation in the brain (48.1 ± 7.5%), with lesser concentration in the kidneys (12.1 ± 3.2%), heart (7.3 ± 0.8%), spleen (7.3 ± 5.5%), and least in the liver (3.02 ± 1.6%). However, there appeared to be no detectable accumulation in the lungs post‐intracarotid injection. This finding contrasts with earlier work carried out via IV tail vein injection, suggesting that the target organ is critically dependent on the site of injection, which is consistent with prior RBC hitchhiking reports.^[^ ^28^, ^29^, ^30^, ^31^ ^]^ The major innovation reported herein reveals the capacity for IL–NPs to hitchhike onto the RBCs post‐injection, while earlier work required the removal of RBCs for NP attachment ex vivo.^[^ ^28^, ^29^, ^30^, ^31^ ^]^ This is possible given that the IL coating imbues stealth properties onto the NP, allowing it to navigate the plasma and serum proteins to contact other blood components, even outperforming poly(ethylene) glycol.^[^ ^26^ ^]^

Once biodistribution was determined with DiD, a new set of healthy, 8‐week‐old, female, Sprague Dawley rats with in‐dwelling carotid catheters received arterial infusions (under the same conditions) with either empty, or ABC‐encapsulated IL‐coated PLGA NPs (n = 3/group). Sites of regional distribution of abacavir cargo were then evaluated within the brain (at the same 6‐hour endpoint). Immediately following exsanguination, brain subregions (i.e., cerebral cortex, hippocampus, striatum, hypothalamus, midbrain, cerebellum, and interbrain) were grossly dissected, subsequently homogenized, and processed for assay via ^1^H‐NMR spectroscopy to identify areas of selective ABC accumulation. As illustrated by both Figure 3F,G, a broad range of proton peaks were found in the filtrate corresponding to those of the IL‐PLGA NPs (such as the methyl or CH_2_ group off the anion alkyl chain respectively at 0.9 and 1.5 ppm, or the protons off the trans‐2‐double bond between 6.5‐7.5 ppm), albeit shifted due to the NP degradation process during extraction (when compared to intact IL‐NPs in Figure S4C,E, Supporting Information). While also slightly shifted due to the co‐solvent composition during extraction, abacavir's signature singlet proton peak is clearly distinguishable from the baseline at 8.1 ppm (Figure S4F,G, Supporting Information) in Figure 3G, when compared to the empty cargo IL‐NP delivery (Figure 3F). Abacavir was observed to accumulate most greatly in the cerebellum, interbrain, striatum, and midbrain regions, with lesser (but considerable) delivery to the hippocampus, cerebral cortex, and hypothalamus regions (Figure 3G). It seems likely that the intracarotid path of microvascular distribution contributed to this pattern of particulate accumulation, with IL‐ PLGA NPs shearing off from RBCs and subsequently crossing the BBB.^[^ ^41^, ^42^ ^]^ Interestingly, as microglial populations are vastly diverse throughout these brain subregions, the potential for deep and comprehensive microglial targeting during HIV can be possible with such a distribution.^[^ ^43^ ^]^

### 2.4 IL‐PLGA NPs Enter the Brain by Shearing Through Blood Vessels and Traffic to Microglia for Selective Uptake

**Figure 4:**
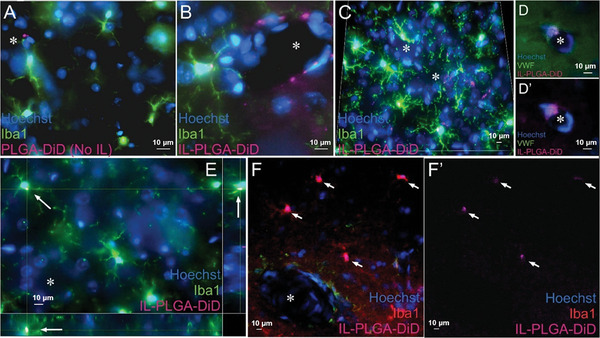
IL‐PLGA NPs enter the brain by shearing through blood vessels and are uptaken by microglia in the caudate/putamen. A) Bare PLGA NPs sporadically in presumed endothelial cells at the boundary of small blood vessels. B,C) IL‐PLGA NPs in parenchymal Iba‐1^+^ microglia and around blood vessels in presumed endothelial cells. C) DiD signal from IL‐coated NPs observed in every microglial soma captured in the field and in several suspected endothelial cells surrounding apparent blood vessels. D,D’) Labeling with von Willebrand factor confirming presence of DiD in endothelial cells. E) Z‐stack imaging supports intracellular localization of DiD in microglia (see bottom and right orthogonal views for virtual cross‐section). F,F’) Frequent uniform DiD co‐localization to microglia next to large vessels. ^*^ Indicates blood vessel. Arrows localize DiD in panels E, F, and F’. Scale bars = 10 microns in every panel.

Bare PLGA DiD NPs were only sparingly identified at the boundary of some small blood vessels in the brain via confocal microscopy (**Figure** **4**A). In contrast, magnitudes‐greater IL‐coated PLGA DiD NPs were observed in blood vessel boundaries where they initially co‐localized to presumed endothelial cells and nearby microglia (Figure 4B), supporting the RBC hitchhiking‐BBB shear theory. However, throughout the caudate/putamen, IL‐PLGA DiD NPs were observed in the soma of parenchymal Iba‐1^+^ microglia and presumed endothelial cells (Figure 4C,F,F’). The identity of endothelial cells was confirmed via co‐localization of DiD with von Willebrand factor‐positive cells (Figure 4D–D’). To confirm microglial uptake versus membrane‐only adsorption, virtual cross‐sections of Z‐stacked images support the notion that DiD signal is located in the intracellular fraction of Iba‐1^+^ cells (Figure 4E).

**Figure 5:**
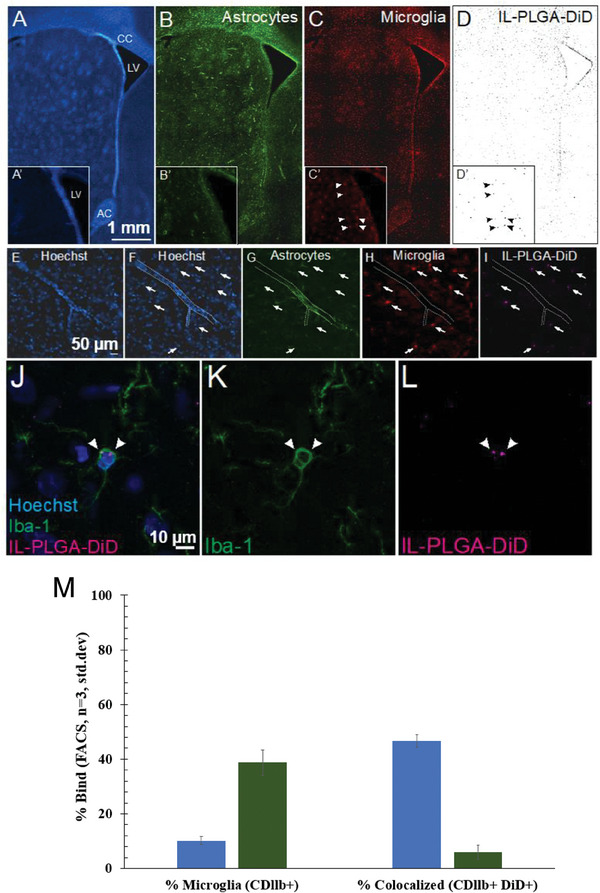
IL‐PLGA DiD NPs in the brain co‐localize selectively with microglia in vivo. A–D) Co‐localization of DiD‐loaded IL‐NPs (D) with microglia (C), but not astrocytes (B), is demonstrated in the parenchyma of the rat dorsal striatum (inset shows the head of the caudate across panels). E–I) A blood vessel (outlined in E‐F) reveals DiD (I) co‐localization with microglia (H), but not astrocytes (G) (arrows localize DiD signal across panels). J–L) Somal expression of DiD is evident in microglia. M) Fractional gated representation of FACS quantification (CDllb^+^ versus CDllb^+^DiD^+^) of isolated and purified microglia shows high co‐localization with IL‐ PLGA DiD NPs (blue) versus saline background (green), the latter of which was only CDllb^+^ (n = 3 internal repetitions of n = 1 brain extract/group ± standard deviation). CC = corpus callosum, LV = lateral ventricle, AC = anterior commissure. Scale bar = 1 mm in Panels A‐D, 50 microns in Panels E‐I, 10 microns in Panels (J–L).

To both qualitatively and quantitatively confirm IL‐ PLGA NP selectivity for cells comprising the HIV reservoir, brain cryosections (40 µm) were collected from rats (post IL‐NP infusion, used above) and co‐labeled for protein markers of astrocytes (GFAP; **Figure** **5**B) and microglia (Iba‐1; Figure 5C) with a Hoechst nuclear counterstain (Figure 5A). Widefield images (10×) of the caudate/putamen within the rat dorsal striatum, a region of dense HIV viral load in the human brain,^[^ ^44^ ^]^ demonstrated an apparent co‐localization of DiD signal (Figure 5D) with Iba‐1 (Figure 5C) (see insets). A transverse cross‐section of a blood vessel is seen (Figure 5E–I; 20×) with the astrocytic component of the blood‐brain barrier visualized (Figure 5G). Surrounding microglia (Figure 5H) are observed to co‐localize with DiD signal (Figure 5I).

To ensure that spectral bleed‐through from the near‐infrared channel was not accounting for co‐localization, Iba‐1 was also assessed with an eGFP secondary antibody and DiD signal was confirmed to co‐localize with Iba‐1^+^ cells via confocal microscopy (Figure 5J–L). Importantly, when microglia were extracted, isolated, and purified from whole Sprague‐Dawley rat brains (at the 6‐hour endpoint) treated with saline or IL‐PLGA DiD NPs (n = 1/group with n = 3 internal repetitions/group), FACS indicated 46.5 ± 2.3% of microglia were DiD^+^ (Figure 5M; Figure S8, Supporting Information), confirming selective microglial uptake identified by confocal imaging.

## 3 Conclusion

We report a novel and highly‐effective strategy for nanoparticle delivery to the brain through the development of bioinspired IL coatings that enable spontaneous hitchhiking onto red blood cells post‐injection. In addition to tissue specificity, the IL coating also shows preferential uptake into microglia in vivo. We also show the successful encapsulation of the ART, abacavir, with in vitro assays indicating that the drug retains efficacy, the IL‐NPs are non‐toxic to PBMCs, and the IL coating significantly increases uptake of the NPs into cells. In all, bioinspired IL coatings are a promising new technology that could make delivery of a variety of therapeutics into the brain feasible and effective. Future studies will focus on determining efficacy in larger animal models, such as macaques, and in disease models, as well as safety and immune profiling.

## 4 Experimental Section

### Materials

Choline Bicarbonate cation (Sigma–Aldrich, #C7519‐500ML) and Trans‐2‐Hexenoic acid anion (Sigma–Aldrich, Cat # W316903‐1KG‐K) were obtained from Sigma–Aldrich. Neat synthesized ionic liquid (IL) was characterized by ^1^H NMR (400 MHz Bruker Ascend) in *DMSO‐D_6_* (99.96 atom% D) (Sigma–Aldrich, Cat # 156914‐10) and by Karl Fischer Titration for water content (Metrohm Coulometer Cat # 899). Resomer RG 504 H, Poly (D, L‐lactide‐co‐glycolide) 50:50 (PLGA) (acid terminated) was obtained from Sigma–Aldrich (Cat # 719900). To study biodistribution of the NPs, the hydrophobic far‐red dye 1,1′‐Dioctadecyl‐3,3,3′,3′‐Tetramethylindocarbocyanine, 4‐Chlorobenzenesulfonate Salt solid (DiD) was used (ThermoFisher, Cat # D7757), while HIV drug cargo encapsulation was performed with abacavir sulfate (ABC) (Cayman Chemical, Cat # 14746). HPLC‐grade Acetonitrile (Sigma–Aldrich, Cat # 34851‐4) was used as the organic phase of nanoprecipitation, with the aqueous phase consisting of Ultrapure MilliQ water (Cat # Milli‐Q IQ 7000). After cold nanoprecipitation and solvent evaporation in a 100 mL round bottom flask (Sigma–Aldrich, Cat # Z414492) on a nine‐plate magnetic stir plate, NPs were cold‐centrifuge filtered (Thermo Scientific Sorvall Ultracentrifuge, Cat # ST8R) using a 30 kDa MWCO Amicon Ultra‐4 filter (EMD Millipore, Cat # UFC803024). For IL‐PLGA NP NMR quantification and analysis, Deuterium Oxide (D_2_O) (99 atom.% D) solvent was used as the aqueous phase (Sigma–Aldrich, Cat# 435767‐1KG). Neat tetramethylsilane (TMS) (density = 0.648 g mL^−1^, MW 88.22 g mol^−1^) NMR internal standard for relative IL and ABC drug quantification was obtained from Sigma–Aldrich (Cat # 87920‐25ML). For NP physiological use, 1X Phosphate‐Buffered Saline (PBS, pH 7.4.) was obtained from Gibco (Cat # 10010072), and 0.9% USP‐injection‐grade sterile saline from Fisher Scientific (Cat # NC9054335). Homogenization studies were performed with an IKA 5G homogenizer (IKA, Cat # 0003304000), 50% BioXtra Trichloroacetic acid (Sigma–Aldrich, Cat # T9159‐500G), 5 m NaOH (Sigma–Aldrich, Cat# 221465‐500G), 1x Tris‐Buffered Saline (TBS) (Cell Signaling, Cat # 12498), and Fisherbrand pH meter (Accumet AB150). Pierce RIPA Lysis Extraction Buffer was acquired from Thermo Scientific (Cat # 89901). Triton‐X (Cat # 807423) was obtained from BM biomedicals LLC, 0.1% BSA (Cat # A6003) from Sigma), trypan blue (Cat # 15250‐061) from Gibco, and BSA (Cat # A6003) from Sigma. anti‐Iba 1, Rabbit, (Cat # 019–19741) was obtained from Wako Chemicals USA Inc. Anti‐Glial Fibrillary Acidic Protein, Clone GA5. Alexa Fluor 488 goat anti‐mouse IgG, (Cat # A11002) and Texas Red Goat anti‐Rabbit IgG (Cat # T2767), and Hoechst (Cat # 33342) were sourced from Invitrogen. Goat Serum (Cat # D204‐00‐0050) was obtained from Rockland Inc. Condition media contained RPMI 1640 (Cat # 22400‐105), 10% FBS heat‐inactivated (Cat # SH30910.03), 1% Pen/Strep, (Cat # 15140‐122), 1% L‐Glutamine (Cat # 17–605E) from Fisher Scientific. Human Astrocyte Primary Cell culture (Cat # 36058‐01) and Human Microglia Primary Cell culture (Cat # 37089‐01) were sourced from Celprogen. PBMC cells (Cat # SER‐PBMC‐P‐F. Lot PBMC070721ABCDE) were obtained from Zen Bio Inc. HIV‐1 virus was obtained from the NIH AIDS reagent program (HIV‐1 Ba‐L virus, Cat # 510, Lot # 150058). p24 antigen assay (ELISA, Cat # 5447) was obtained from ABL Inc.

### Ionic Liquid Synthesis and Characterization

Choline 2‐hexenoate (Choline Trans‐2‐Hexenoic Acid, CA2HA 1:2) was synthesized as previously published.^[^ ^45^ ^]^ Briefly, choline bicarbonate (80% in water) was combined dropwise with 98% pure trans‐2‐hexenoic acid at a 1:2 molar ionic ratio at 40 °C, while stirring in an oil bath overnight. CA2HA 1:2 IL was then rotary evaporated to remove any residual solvent at 15 mbar for 2 h at 60 °C, and then vacuum dried at −760 mmHg for 48 h at 60 °C. The product (332.5 g mol^−1^, density 1.66 g mL^−1^) was massed by analytical balance to calculate yield (82.6%), evaluated by Karl Fischer to measure IL water content (1.68% (wt/wt)), and assayed by NMR to evaluate purity and chemical identity: ^1^H NMR (300 MHz, DMSO) δ 6.41–6.29 (m, 2H), 5.50 (dt, *J* = 15.6, 1.5 Hz, 2H), 3.62 (dq, *J* = 5.0, 2.6 Hz, 2H), 3.20 (dd, *J* = 6.3, 3.7 Hz, 2H), 2.89 (s, 9H), 1.83–1.72 (m, 4H), 1.10 (p, *J* = 7.3 Hz, 4H), 0.58 (t, *J* = 7.3 Hz, 6H).

### NP Synthesis

Bare PLGA and IL‐PLGA NPs were synthesized as previously published.^[^ ^45^ ^]^ Briefly, to make bare PLGA DiD NPs, a fluorescent dye stock solution of 1 mg mL^−1^ DiD/ACN was prepared, and combined with an organic phase stock solution of 1 mg mL^−1^ PLGA/ACN at 2% by weight of the polymer. To make 1 mg of NPs, the organic phase (1 mL) was then combined slowly dropwise in a 100 mL round bottom flask at 1200 RPM with an aqueous phase of either 3 mL Milli‐Q water or D_2_O water (for chemical characterization), and allowed to stir uncovered on a magnetic plate for 3 h in the dark at 1200 RPM and 25 °C. At 800 RPM, 10 mg neat IL/mg PLGA was then added to the solution in the form of a liquid droplet by gravity in the center of the stirring vortex, and allowed to stir for two more hours at 25 °C in the dark (1 h at 800 RPM, 1 h at 900 RPM), before 30 kDa MWCO centrifuge filtration at 4 °C and 2500 RPM for 1 h. To investigate the role of the choline cation and trans‐2‐hexenoate anion in both self‐assembly of the IL coating on PLGA NPs, as well as for RBC hitchhiking, IL‐NP synthesis was repeated as previously described, however adjusted with the following formulation parameters, added at 800 RPM: 1) 5.8 mg of purified 2HA anion and 4.2 mg of choline bicarbonate (to form CA2HA 1:2 in situ), 2) 5.8 mg of purified 2HA anion, and 3) 4.2 mg of choline bicarbonate. Bare PLGA NPs were 30 kDa filtered, however more gently at the same temperature, between 1500–2000 RPM. All filtrates were then collected and resuspended to 1 mg mL^−1^ in either Milli‐Q water, D_2_O, 1x PBS pH 7.4, or 0.9% USP‐grade saline for the respective DLS or NMR characterization, or biological application. Samples were then stored at 4 °C in the dark for up to 2 weeks, although used within the first week of synthesis.

*NP Synthesis with Abacavir (ABC) Cargo*: Bare PLGA and IL‐PLGA NPs were synthesized again as described above, however with a different preparation of the HIV drug cargo. A stock of abacavir (ABC) was prepared in ACN at a 1 mg mL^−1^ concentration and kept cold at −20 °C. The drug stock was then combined with the organic phase again at 2% (wt ABC/ wt PLGA), however scaled respectively by the molecular weight of ABC (286.3 g mol^−1^) versus DiD (1052.08 g mol^−1^). After vortexing to incorporate, the organic phase was then stored at −20 °C for 2 h before use. When performing nanoprecipitation, round bottom flasks on the magnetic stir plate were surrounded by reusable ice blocks to ensure a cold environment for the stirring solution. After capping, both PLGA and IL‐PLGA NPs were filtered as described above and stored at 4 °C for the same time frame.

### NP Size and Surface Charge Characterization

For both 1 mg mL^−1^ filtered DiD and ABC‐encapsulated PLGA NPs, a Malvern Zetasizer (Pro Blue) was used (software version 3.0) for Dynamic Light Scattering (DLS) measurements. An optimized calibration time of 40 s was used for both samples after a 120 s optimization time was used to evaluate the scattering profile of ABC‐encapsulated NPs, with a fluorescent filter engaged for DiD NPs. A dip cell (Malvern ZEN1002) was used for zeta potential surface measurements while a DTS 0012 polystyrene cuvette was used for size determination. All samples were evaluated, at minimum, in triplicate.

### Cargo Encapsulation Efficiency (EE)

To validate that the fluorescence of the tracked NPs in vivo was derived from cargo encapsulation and not from free DiD dye leakage, encapsulation efficiency, absorption by UV–vis spectroscopy, and fluorescence emission were measured, as described below.

*Relative Encapsulated Fluorescence (DiD)*: In a 96‐well black opaque plate, 200 µL of synthesized 2% DiD 1 mg mL^−1^ PLGA and CA2HA 1:2 coated PLGA NPs were plated per independently synthesized replicate (n = 3) and compared to 200 µL buffer used for suspension and 200 µL of the same 2% organic phase stock used to synthesize the resulting particles. The plate was read from the top and height adjusted to optimize for the volume of samples in the well, and then fluorescently measured at 640 nm excitation and 670 nm emission (BioTek Synergy H1 plate reader). Fluorescence of the buffer was subtracted from all samples, and then samples were divided by fluorescence of the 2% DiD organic phase stock (100%). Standard deviation was used to measure the spread of DiD encapsulation among the particles.

*DiD‐Nanoparticle Absorbance by UV–vis Spectroscopy*: Using Hellma Herasil quartz absorption cuvettes (standard cells, parameter 260–2,500 nm spectral range, pathlength 10 mm, chamber volume 3,500 µL), the UV–Vis absorption spectrum profile of 1 mg mL^−1^ of bare PLGA DiD NPs & CA2HA 1:2 PLGA DiD NPs in 0.9% NaCl were measured from 200–800 nm, alongside controls of 0.02 mg DiD in ACN (free dye in solvent) & 0.02 mg DiD in 1 mg mL^−1^ PLGA:ACN organic phase (covalently‐bound DiD‐PLGA in ACN). The absorption spectra of the prepared nanoparticles (and reference solutions) were measured using a double reference Cary 5000 spectrophotometer (Cary 5000 UV–vis‐NIR, Agilent). ACN and NaCl solutions were respectively used as reference account for any possible background absorbance and baseline correction was done to remove the same from interfering with the samples. The absorption intensity of each sample was normalized to compare the absorption profile of CA2HA 1:2 PLGA DiD NPs against PLGA DiD NPs and DiD dye to see the effect of IL coating on the absorption features of the DiD‐loaded PLGA NPs.

*DiD‐Nanoparticle Fluorescent Emission by Fluorimetry*: The fluorescence emission profile of 1 mg mL^−1^ of bare PLGA DiD NPs & CA2HA 1:2 PLGA DiD NPs in 0.9% USP‐grade NaCl were measured on a Horiba FluoroMax SpectroFluorimeter (System #3657, model QM‐8075‐21‐C with PTI ASOC‐10) from the far‐red to NIR region (650–800 nm), alongside controls of 0.02 mg DiD in ACN (free dye in solvent) & 0.02 mg DiD in 1 mg mL^−1^ PLGA:ACN organic phase (covalently‐bound DiD‐PLGA in ACN). The excitation wavelength for DiD was calibrated to 635.6 nm, with an excitation slit width resolution of 5 nm and an emission slit width of 5 nm in a quartz cuvette of path length 10 mm (step size: 0.5 nm, integration: 1 s). The excitation wavelengths were generated by passing white light through a dual‐grating system and the photons were collected through a photomultiplier tube. The fluorescence emission spectra of all the samples were exported as a .txt file, converted to .csv, and baseline subtracted by their respective solvent background and plotted in an overlay fashion for the better understanding of the trend of DiD emission profile among different DiD NP samples with respect to DiD stock.

*32 h Release Profile of DiD dye from PLGA DiD NPs and CA2HA 1:2 DiD NPs in Saline*: Adapted from a similar protocol,^[^ ^46^ ^]^ synthesized bare PLGA (n = 3) or CA2HA 1:2 PLGA DiD NPs (n = 4) (1 mg mL^−1^, in 0.9% USP‐grade saline) were suspended inside a tied Snakeskin dialysis tubing membrane pre‐flushed with the same 0.9% saline buffer (3.5 kDa, Thermo Scientific, Cat # 88242), in 5 mL of the same saline buffer in a 25 mL glass scintillation vial. The membrane containing DiD NPs underwent magnetic stirring at 400 RPM and 37 °C in the dark, and 1 mL of buffer was sampled and replaced at the following timepoints (in hours): 0 (sampled immediately after stirring began),1,2,3,4,5,16,21,24, and 32 h. At each timepoint, 1 mL of sampled buffer was distributed into four wells in a 96‐well black plate (Greiner Fluorotrac) at 250 µL per well. The fluorescence of each well was read at 640 excitation, 670 emission via a Cytation Five imaging fluorescent plate reader (Agilent technologies, Model CYT5MF), added together, and multiplied by five to account for the scaling of the release buffer. The total scaled fluorescence at each timepoint was then summed cumulatively across timepoints and divided by a DiD expected yield control (100%). This control was prepared by quickly flushing a 1 mL of a replicate of PLGA or CA2HA 1:2 PLGA DiD NPs (1 mg mL^−1^) through a saline pre‐soaked snakeskin membrane (to account for any immediately free DiD capture) and then distributed across four wells, which was added, to serve as the “100% DiD yield” for each sample release study.

*Quantitative Encapsulation of ABC (Proton Nuclear Magnetic Resonance Spectroscopy (^1^H NMR)*: Bare PLGA and CA2HA 1:2 PLGA NPs were synthesized in deuterium oxide as described previously, and then resuspended to 500 µL (2 mg mL^−1^) to quantitatively measure composition of the IL coating on the surface of the PLGA NPs with 20 uL Tetramethylsilane (TMS) internal quantification standard: ^1^H NMR (400 MHz, D_2_O) δ 6.85 (dt, *J* = 15.8, 7.0 Hz, 27H), 5.85 (dt, *J* = 15.7, 1.5 Hz, 28H), 4.78 (d, *J* = 1.3 Hz, 7140H), 4.09–4.02 (m, 27H), 3.81–3.71 (m, 21H), 3.64 (dd, *J* = 11.8, 4.5 Hz, 45H), 3.60–3.47 (m, 73H), 3.25 (s, 136H), 2.72 (d, *J* = 1.3 Hz, 14964H), 2.19 (qd, *J* = 7.2, 1.6 Hz, 69H), 1.56–1.46 (m, 47H), 0.91 (t, *J* = 7.4 Hz, 96H), 0.00 (s, 12H).

To break open the NPs, bare PLGA NPs, and IL‐PLGA NPs were first sonicated at 60 °C for 30 min to destroy the outer IL coating integrity, evidenced by a phase separation of cloudiness in the IL‐PLGA NPs. To next dissociate the IL coating from the polymer core, all particles were vortexed at 25 °C on the highest setting for 10 min (in 2‐min intervals). The particles were then stored at −80 °C for 2 min to protect the ABC drug while inducing a bulk phase separation, and then vortexed at the highest setting for 2 min at 25 °C. Lastly, to extrude the abacavir from the polymer core, all NPs were 30 kDa MWCO centrifuge‐filtered for 1 h at 4500 RPM at 40 °C. Filtrate smaller than 30 kDa was collected at the bottom of the tube and stored at −20 °C, concentrated (2x) with D_2_O and evaluated for presence of drug by ^1^H NMR at 400 MHz at the highest scan rate (proton64).

As the appearance of drug peaks were very small compared to the IL, 2 µL of TMS was then used in particular to obtain a relative understanding of the size of the small emerging peaks. TMS at 0 ppm was then integrated to 12 protons and compared to a small new singlet peak at 8.5 ppm indicating emergence of a unique proton proximal to ABC's cyclic nitrogenous base structure. Detection of other scattered protons in the cyclic structures in between IL peaks, possibly indicates protective effects of the IL upon ester degradation of the polymer. Representation of encapsulated ABC dose was then calculated:

0.002 mL TMS (0.648 g mL^−1^)  =  0.0013 g TMS

0.0013 g TMS/88.22 g mol^‐1^ TMS  =  0.0000146 mol TMS

0.0000146 mol TMS (≈ 0.03 integral ratio, from between 8.5 and 0 ppm peaks) ≈ 4.38 x 10^−7^ mol ABC

4.38 × 10^−7^ mol ABC (286.3 g/mol ABC) ≈ (0.000125 g ABC/2) (where µg total ≈ 1 mg mL^−1^)

≈62.7 µg mL^−1^ ABC encapsulated

### Scanning Electron Microscopy (SEM)

Bare PLGA and CA2HA 1:2 PLGA NPs without cargo were synthesized to evaluate topological and morphological differences on the nano‐scale. Briefly, nanoparticles were prepared as described above, and then 10 µL of respective solution was dropcast onto plasma‐cleaned 9.5 mm × 9.5 mm cylinder aluminum SEM sample stubs (JEOL, Cat # 10‐005110‐50). Samples were allowed to dry overnight at 4 °C and ambient conditions in an airtight environment to prevent dust deposition. The following day, all samples were then sputter coated with Palladium to enhance imaging contrast (16.5 mm, 35 mA, 200 s). Imaging was performed on the same day with a JSM‐7200 FLV Field‐Emission Scanning Electron Microscope (FESEM).

### In Vitro Studies

*RBC Hitchhiking of IL* versus *IL Components on PLGA DiD NPs in Whole Blood*: Bare PLGA DiD NPs, CA2HA 1:2 PLGA DiD NPs, CA+2HA 1:2 PLGA DiD NPs, Choline bicarbonate PLGA DiD NPs, and 2HA PLGA DiD NPs, were evaluated for RBC hitchhiking in whole blood to investigate whether molecular assembly of cation and anion alone on PLGA DiD NPs were sufficient to achieve comparable levels of formulation stability and RBC hitchhiking. To test each of the formulations in the harshest conditions, 900 µL aliquots of adult female human whole blood (Heparin‐anticoagulated, Bio‐IVT, USA) were first treated on ice 1:10 (v/v) (≈[100:1] NP:RBC ratio) with all NP formulations (0.5 mg mL^−1^ in 0.9% saline) and saline control (n = 3). Treated whole blood then was shaking‐incubated for 20 min at 500 RPM and 37 °C.^[^ ^26^, ^27^, ^45^ ^]^ RBCs were then isolated from whole blood by density‐gradient via centrifugation at 1000xg, 10 min, at 4 °C, and then washed to the original volume of whole blood with saline three times at 4 °C at 200xg for 5 min each wash. Final RBC aliquots were then brought up to 1 mL, and 200 µL of that solution was diluted to 2 mL with saline buffer to be read by fluorescence activated cell sorting (FACS) (Thermo Fisher Scientific, Attune Nxt Flow Cytometer). RBC singlets were gated via SSC‐A versus FSC‐A scattering and FSC‐H versus FSC‐A scattering, and then evaluated against the RL1 laser (far‐red) to identify % RBC population with at least one or more DiD NPs/RBC (quadrant‐shifted from saline control RBC population). Background noise from saline was subtracted from sample measurements, and background‐subtracted sample measurements were then normalized to bare PLGA DiD NPs (set to 1) to evaluate fold‐greater RBC hitchhiking.

*MCT‐1 Inhibition on the Role of 2HA Affinity to RBCs in Whole Blood*: RBCs were isolated from whole BALB/c gender‐pooled mouse blood (k2‐EDTA anticoagulated, Bio‐IVT, USA). First wash to isolate RBCs was performed at 1000xg, 10 min, 4 °C. Isolated RBCs were then washed at 200xg, 5 min, 4 °C, three times to eliminate pre‐hemolytic events. Washed RBC pellet was transferred to make several [1:50 v/v] RBC stocks in 0.9% saline buffer. In triplicate, 396 µL of RBC stock was pre‐treated with either 100 µm *α*‐Cyano‐4‐hydroxycinnamic acid (CHC)^[^ ^47^, ^48^, ^49^ ^]^ (MCT‐1 Inhibitor, Sigma–Aldrich cat. # 476870) or 0.4 µm cytochalasin B^[^ ^50^, ^51^ ^]^ (GLUT inhibitor, Cayman Chemical Company, cat. # 11328), in cell culture‐grade DMSO. Inhibitors and RBCs were mixed together at 200 RPM and 25 °C for 30 min., before resting overnight at 4 °C to slowly fully saturate receptors with inhibitor. Inhibition of the GLUT membrane transporter served as an internal control to account for non‐selective binding during membrane inhibition/damage as well as to account for indirect biological cascades as a result of impairing normal glycolytic pathways during MCT‐1 inhibition.^[^ ^52^ ^]^

To additionally evaluate the impact of RBC membrane dynamics on hitchhiking alongside MCT‐1, 396 µL of RBC stock was treated with either 0.1% (wt vol^−1^) Triton (for lipid extraction) and 0.25% (wt vol^−1^) Glutaraldehyde (for protein crosslinking) in 0.9% saline buffer, in triplicate, by mixing for 1 min at 200 RPM at 25 °C, before incubation at 4 °C for 15 min. This was experimentally determined to be enough time to visually observe the cells rigidify without inducing notable hemolysis. After their respective incubation conditions, all treatments were then washed 3x to remove all inhibitor and residual hemolytic events (1000xg, 10 min first wash; 2nd, and 3rd washes were for 5 min at 4 °C) and replaced to original volume with saline buffer. All inhibited RBCs were then treated at a [100:1] NP:RBC ratio with controls, PLGA DiD NPs, and CA2HA 1:2 PLGA DiD NPs (1 mg mL^−1^ in saline), via shaking‐incubation at 37 °C and 500 RPM for 20 min.

For MCT‐1 inhibited RBCs, unbound or weakly‐bound NPs were washed off 3x at 4 °C, 5 min per wash. The first two washes were at 200xg, the third wash to increase shear of residually‐bound NPs was at 500xg. For GLUT control transporter and membrane dynamics‐inhibited RBCs, NPs were washed off via centrifugation 3x at 1000xg, 10 min, 4 °C per wash. The rationale for the difference in washing conditions was that NPs mechanically adsorbed to lipids or proteins (or don't have affinity to GLUT), would require constant mechanical stress to remove NPs per wash. For all treatments, after the first wash, total supernatant was collected and added 200 µL per well into a 96‐well black plate. After the first wash, RBC pellet was brought up to 200 µL with saline per wash, so that the supernatant added to each well for wash two and three were 200 µL per well. After the final wash, RBC pellet was brought to 200 µL with saline and all was added into a well. Finally, all samples were read for DiD fluorescent emission in a Greiner Fluorotrac 96‐well black plate (Greiner Bio‐One, Cat #655076), on a fluorescent plate reader (Biotek, Synergy H1) at 640 ex/670 em. As no sample was discarded, fluorescence of all washes and RBCs was added to the total possible NPs (100%) to calculate NP distribution among all the fractions.

*HIV Expression and Cytotoxicity*: Three independent batches of bare PLGA and CA2HA 1:2‐coated PLGA NPs were prepared with abacavir (ABC) as above but in sterile Milli‐Q water and re‐suspended to 1 mg mL^−1^ with sterile 1x PBS pH 7.4. As ≈60 µg mL^−1^ of drug was estimated by quantitative NMR, PBMC cells were stimulated with 10 µg mL^−1^ of PHA for 4 days. Stimulated PBMC cells were harvested at 400xg, 25 °CR for 10 min. The cells at 2  ×  10^6^ cells per mL were used for each experimental condition and infected with 1 ng mL^−1^ of HIV‐1 BaL^+^ in presence of 3 ng mL^−1^ of IL‐2 and 2 µg mL^−1^ of polybrene. PBMC cells were treated in the absence or presence of nanoparticles at 10 µm final concentration. An equivalent concentration of free ABC (10 µm) was used to treat PBMC cells in parallel with NPs. The treated PBMC cells were harvested at 3‐, 7‐, and 10‐days post infection. The cell viability was determined by trypan blue at each harvesting day and an aliquot of condition media was saved for further analysis on p24 antigen assay. The PBMC cells were treated freshly with NPs or ABC at each time point, except day 10. The p24 antigen ELISA assay was performed according to manufacture protocol on experimental condition media collected on 3, 7, 10 days post HIV‐1 infection for quantification of HIV‐1.

*Immunohistochemistry*: Primary human astrocyte and primary human microglia (see Experimental Section) were co‐cultured at 25000 and 6000 cell density in six well plate respectively, or primary human microglia were cultured alone at the same seeding density. The cells were maintained according to manufacturer's protocol (Celprogen Inc, Torrance, CA). Cells were treated with and without PLGA‐DiD NPs, and IL‐PLGA‐DiD NPs at 10 µm final concentration for 24 h. At the end of incubation, the cells were fixed with 4% PFA for 10 min (25 °C), and then permeabilized (0.1% Triton‐X, 0.1% BSA in PBS) for 30 min at 25 °C. Cells were washed 3x with PBS and then blocked with blocking buffer (1% BSA, 1% normal goat serum in PBS) for 1 h at 25 °C. The cells were probed with mouse anti‐GFAP (for astrocytes) and/or rabbit anti‐Iba1 (for microglia) both at 1:200 dilution, overnight at 4 °C. Cells were washed 3x with 1x PBS pH 7.4, and incubated with Alexa 488 goat anti‐rabbit and/or Alexa 488 goat anti‐mouse secondary antibodies (1:500 dilution), and Hoechst for nuclear counterstain for 1 h at 25 °C. Intracellular DiD accumulation was visualized using a TI2‐E motorized, inverted microscope (Ti2‐S JS, Nikon instrument Inc.).

### In Vivo Studies

All animal experiments were carried out under the supervision of the University of Mississippi IACUC under protocol #21‐004.

*Injection Procedure*: Female Sprague‐Dawley rats (≈75 days of age) with in‐dwelling, intra‐carotid catheters were purchased from Envigo (St. Louis, MO, USA). Rats were acclimated to a temperature‐ and humidity‐controlled room in the vivarium at the University of Mississippi for at least 7 days prior to experimental manipulation. Catheter patency was maintained via a flush (50% heparin in saline (IU mL^−1^), 72 h. after arrival). At the time of manipulation, rats received a slow intra‐arterial infusion of 500 µL over 5 min (100 µL min^−1^) of saline, bare PLGA‐NPs encapsulating DiD (1 mg mL^−1^ concentration in 0.9% (wt vol^−1^) USP‐grade saline), IL‐PLGA‐NPs encapsulating DiD (1 mg mL^−1^ concentration in 0.9% (wt vol^−1^) USP‐grade saline), or IL‐PLGA NPs (with and without 60 µg mL^−1^ ABC) in 0.9% (wt vol^−1^) USP‐grade saline.

*Biodistribution*: After cardiac exsanguination (blood collection by cardiac puncture, followed by transcardial perfusion with 1x PBS pH 7.4) at the 6‐hour endpoint to collect all remaining whole blood for flow cytometry detection (Figure S4, Supporting Information) of circulating DiD‐encapsulated NPs (RBC hitchhiking: FACS on isolated RBCs from whole blood performed according to conditions as previously described^[^ ^27^ ^]^), perfused whole blood‐filtering organs were then collected and flash‐frozen in triplicate (in dry ice and ethanol) and stored at −80 °C. All organs were then massed and then homogenized at 10x scale (by mass) in 50% (v/v) Trichloroacetic Acid (TCA) in 1x PBS (pH 7.4) (ie. 1000 mg in 10 mL of buffer). After all tissues were homogenized into a fine solution, homogenates were centrifuged at 4000xg at 4 °C for 10 min. Supernatant was then collected, and stored on ice while each solution was dropwise titrated to pH 8.0 with 5 m NaOH and 1x TBS (range of [1:0.4]–[1:0.6] ratio of supernatant to NaOH, and range of 680 µL–3 mL added 1xTBS, when titrating against each sample). Once neutralized, each sample was vortexed, and then 200 µL was removed from the center of each vial, complemented by a 200 µL calibration curve for DiD fluorescence (0.5–0 mg mL^−1^), and fluorescence of 200 µL of 2% DiD NPs used for the injection study. Fluorescent readings from the wells were then normalized to the frozen mass of each tissue and volume of neutralized buffer solution, then compared to that of the DiD NPs to generate a percent of injected dose (% ID per tissue). Data was represented as average with standard error of mean (SEM) and statistical tests were performed using a paired two‐tailed *t*‐test for two samples at a time (*p* < 0.05).

*Abacavir Detection in the Brain*: In parallel with DiD‐NP injections, Sprague‐Dawley rats injected with IL‐PLGA NPs (either without cargo or with 60 µg mL^−1^ ABC, n = 3) were sacrificed at 6 hours post‐injection, and whole organs were processed, and frozen post‐mortem as described above in *Biodistribution*. In order to assess to areas of biodistribution of abacavir *within* the brain, the following regions were sliced and homogenized in a 1:1 mixture of 1x PBS (pH 7.4) and Acetonitrile constituting a total homogenate volume of 500 µL per sample: striatum, midbrain, interbrain, cerebellum, hypothalamus, cerebral cortex, & hippocampus. To release NPs entrapped within the brain tissue, 500 µL RIPA lysis extraction buffer was first added to each homogenate [1:1 v/v] in a 1.5 mL Eppendorf centrifuge tube and brought to a final volume of 1 mL. All homogenates were then sonicated at 25 °C for 1 hour (with ice cubes added to prevent temperature elevation), followed by vortexing each sample at the highest setting for 10 min (in 2‐min intervals). Next, to assist in rupturing any extracted NPs, brain‐region homogenates were stored at −80 °C for 2 min, vortexed at 2 min at 25 °C, followed by centrifugation at 4 °C and 17850 RPM for 15 min to fully break open and extract any remaining nanoparticle material and/or abacavir cargo from the tissue pellet. Lastly, each brain homogenate was shaken by inversion to resuspend the pellet and supernatant and filtered by 30 kDa centrifugation at 4500 RPM at 4 °C for 1 h to extrude filtrate liquid smaller than 30 kDa through the tissue to the bottom of the tube. A range of 500–600 µL of liquid filtrate was recovered across all samples, which were all delivered into clean NMR tubes and topped 10% (v/v) with D_2_O to be evaluated for presence of drug by ^1^H NMR at 400 MHz with 90% H_2_O /10% D_2_O water suppression.

*Microglial Extraction and FACS*: To confirm microglial‐specific uptake observed in the brain by confocal microscopy, rats injected with either saline (n = 1) or IL‐PLGA DiD NPs (n = 1) were evaluated for quantitative microglial uptake by fluorescence activated cell sorting (FACS) which was followed exactly according to a previously published protocol.^[^ ^53^ ^]^ Briefly, both control and IL‐PLGA DiD NP‐treated rats were perfused with 1x HBSS (Thermo Fisher, Cat # 14175079) 6 hours post‐injection, and extracted whole brains were submerged and frozen at −80 °C in 7 mL of phenol‐red‐free RPMI 1640 buffer (Gibco, Cat # 11835, 500 mL). Whole brains were then homogenized in a 15 mL conical tube until a fine solution in the RPMI buffer, and then treated and layered with 3 mL 100% and 2 mL 70% Percoll (Sigma–Aldrich, Cat # 17‐0891‐02) to isolate microglia via a density gradient post‐ centrifugation (30 min at 550xg and 18 °C). Isolated cells were then washed in 1x HBSS/10 mm HEPES (Gibco, Cat # 15630) via centrifugation for 7 min at 900xg and 4 °C and removed of myelin debris via Myelin Removal beads (Miltenyi Biotec, Cat # 130‐096‐733) and MACS separation system (Miltenyi Biotec, Cat # 130‐042‐108). Purified microglia were then labelled by CDllb^+^ rat microbeads (Cat # 130‐105‐634) via MACS separation system, and then stained for FACS via FITC anti‐rat CD11b/c Antibody (BioLegend, Cat #201805).^[^ ^53^ ^]^ Three internal aliquots were run per sample (NaCl vs IL‐NP) at the lowest flow rate (12.5 µL min^−1^) and 100, 000 count‐rate. Side scatter versus forward scatter (SSC‐A vs FSC‐A) was first used to identify scattering cells, which were gated and examined for singlet scattering (FSC‐H vs FSC‐A) before identifying FITC‐CDllb^+^ microglia (BL1‐A vs FSC‐A) and gating the CDllb^+^ [high] population against far‐red DiD fluorescence (BL1‐A vs RL1‐A).

Confocal Fluorescence: Perfused and cryopreserved rat brains were cryo‐sectioned using a microtome (Leica Biosystems, Cat # CM3050S) at 20‐micron thickness. The slides were washed twice with 1x PBS and permeabilized (with 0.1% Triton‐X, 0.1% BSA in PBS) for 30 min at 25 °C. The permeabilized slides were blocked (1% BSA, 1% normal goat serum in PBS) for an additional 30 min at 25 °C and followed by probing with primary rabbit‐anti‐IBal (1:200 dilution) or anti‐mouse GFP (1:250 dilution) for 90 min at 25 °C. At the end of the incubation, the slides were washed 3x with 1x PBS and followed by incubation with secondary antibodies (Texas Red Goat‐anti Rabbit, 1:500 for Iba1 and Alexa 488 goat anti‐mouse, 1:500 for GFP), 1hr at 25 °C. To stain cellular nuclei, Hoechst stain was used at 1:10,000 dilution. All dilutions represented as (vol/vol). The slides were washed 3x with 1x PBS and mounted with antifade media and kept at 4 °C for further analysis with confocal microscopy.

## Conflict of Interest

J.J.P. acknowledges a business relationship with Nephropathology Associates, PLC dba Arkana Laboratories. Business partners and funders had no role in the design of the study; in the collection, analyses, or interpretation of data; in the writing of the manuscript; or in the decision to publish the results. E.E.L.T., C.M.H., and J.J.P. are named as inventors on provisional patents disclosing the results described here.

## Supporting information

Supporting Information

## Data Availability

Data will be uploaded to FigShare upon acceptance of the article.
