# Supplementary material for: Ionic Liquid Coating‐Driven Nanoparticle Delivery to the Brain: Applications for NeuroHIV
Source: Adv Sci (Weinh). 2024 Apr 4;11(23):2305484. doi: 10.1002/advs.202305484 (PMC11186118; doi:10.1002/advs.202305484)
Supplement: Supplementary file 1 — Supporting Information [file ADVS-11-2305484-s001.pdf]

ADVANCED SCIENCE
Open Access

Supporting Information

for *Adv. Sci.*, DOI 10.1002/advs.202305484

Ionic Liquid Coating-Driven Nanoparticle Delivery to the Brain: Applications for NeuroHIV

Christine M. Hamadani, Fakhri Mahdi, Anya Merrell, Jack Flanders, Ruofan Cao, Priyavrat Vashisth, Gaya S. Dasanayake, Donovan S. Darlington, Gagandeep Singh, Mercedes C. Pride, Wake G. Monroe, George R. Taylor, Alysha N. Hunter, Gregg Roman, Jason J. Paris and Eden E. L. Tanner**

Supporting Information

Ionic Liquid Coating-Driven Nanoparticle Delivery to the Brain: Applications for Neuro-HIV

Christine M. Hamadani[1], Fakhri Mahdi[2], Anya Merrell[1], Jack Flanders[1], Ruofan Cao[2], Priyavrat Vashisth[1], Gaya S. Dasanayake[1], Donovan S. Darlington[1], Gagandeep Singh[1], Mercedes C. Pride[1], Wake G. Monroe[1], George Taylor[1], Alysha N. Hunter[1], Gregg Roman[2], Jason J. Paris[2*], Eden E. L. Tanner[1*]

[1]Department of Chemistry & Biochemistry, The University of Mississippi, University, MS 38677, United States
[2]Department of BioMolecular Sciences, The University of Mississippi, University, MS 38677, United States

*****Addresses for correspondence:

Dr. Jason J. Paris
Department of BioMolecular Sciences
The University of Mississippi
University, MS, 38677
E-mail: parisj@olemiss.edu
Phone: 662-915-3096

Dr. Eden E. L. Tanner
Department of Chemistry & Biochemistry
The University of Mississippi
University, MS 38677, United States
E-mail: eetanner@olemiss.edu
Phone: 662-915-1165

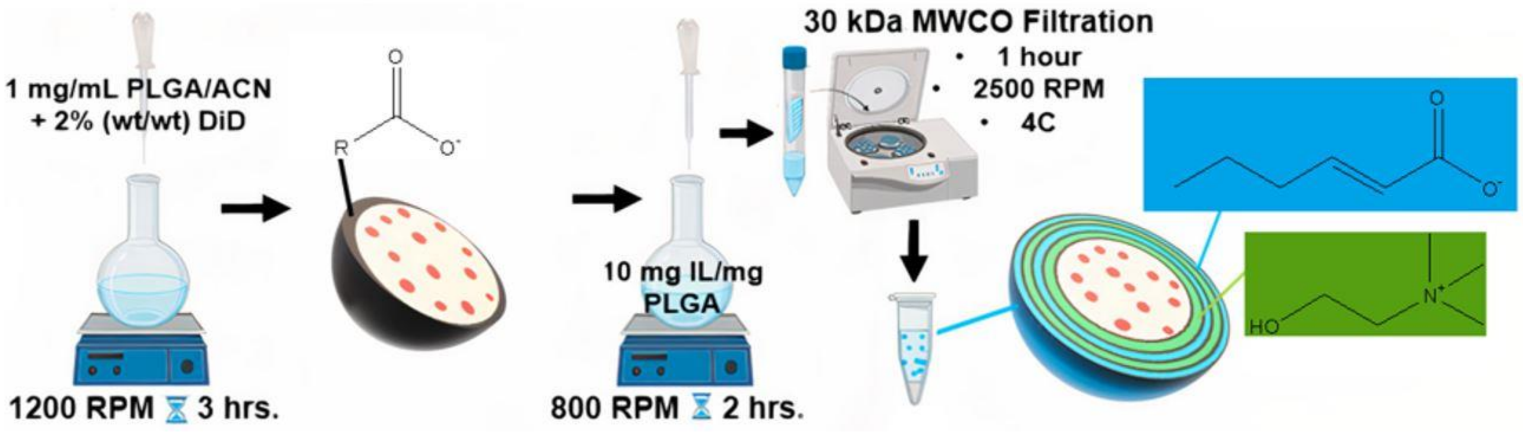

Figure S1. Schematic of IL-NP preparation by nanoprecipitation and solvent evaporation.

Table S1. Summarized DLS Data of Synthesized NPs

Treatment	Size [nm]	Surface Charge [mV]	Polydispersity index [PDI]
**PLGA (empty) (n=4)** [45]	45.1 ± 4.8	-26.6 ± 5.2	0.17 ± 0.06
**IL-PLGA (empty)(n=4)** [45]	142.0 ± 5.6	-43.9 ± 3.9	0.07 ± 0.03
**PLGA (dye) (n=3)**	61.6 ± 1.3	-24.8 ± 0.26	0.11 ± 0.015
**IL-PLGA (dye) (n=4)**	164.6 ± 10.5	-53.0 ± 12.9	0.18 ± 0.03
**CA+2HA PLGA (dye) (n=3)**	171.9 ± 47.0	-59.0 ± 5.9	0.11 ± 0.12
**Choline Bicarb. PLGA (dye) (n=3)**	120.5 ±1.47	-49.8 ±1.7	0.04±0.02
**2HA PLGA (dye) (n=3)**	260.10 ± 55.1	16.1 ± 8.7	0.20 ± 0.14
**PLGA (ABC) (n=7)**	76.4 ± 13.5	-36.9 ± 9.8	0.09 ± 0.04
**IL-PLGA (ABC) (n=5)**	191.5 ± 23.6	-54.8 ± 6.5	0.12 ± 0.07

[45]As previously reported in Hamadani et al., 2022 (Nature Protocols; empty PLGA & IL-PLGA only).

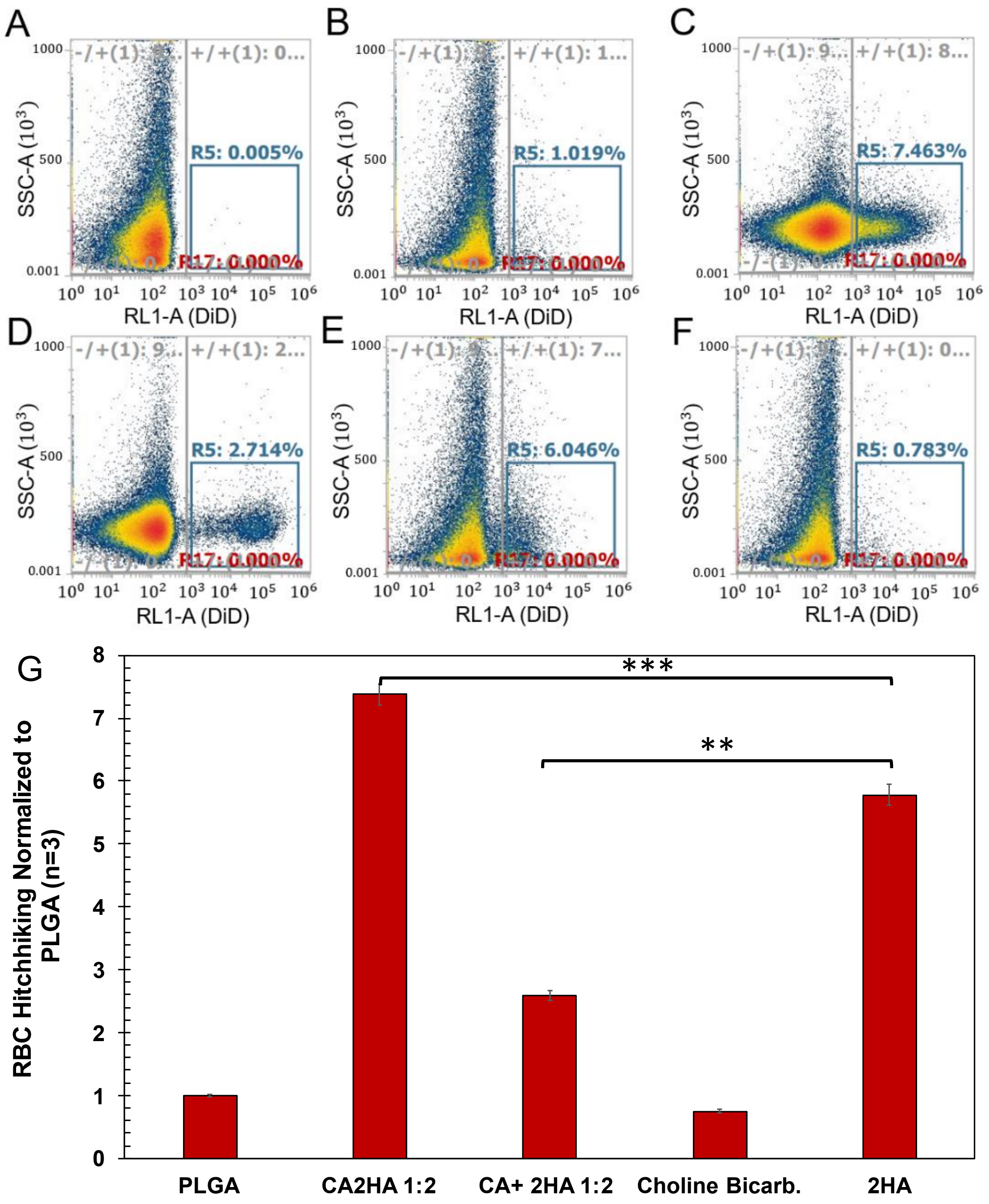

Figure S2. The addition of neat CA2HA 1:2 IL to bare PLGA DiD NPs is essential for maximizing RBC hitchhiking in whole human blood, mediated by trans-2-hexenoate anion, and stabilized by choline during the coating process. Fluorescence Activated Cell Sorting (FACS) profile of RBCs from human adult female whole blood (heparin-anticoagulated) treated with (A) 0.9% saline, (B) bare PLGA DiD NPs, (C) CA2HA 1:2-PLGA DiD NPs, (D) choline bicarbonate and purified trans-2-hexenoate added at a 1:2 molar ionic ratio (CA+2HA 1:2), (E) purified trans-2-hexenoate (2HA) added at the same amount as in (D), (F) choline bicarbonate (choline bicarb.) added in the same amount as in (D). Pictured in (G) is the amount of RBC hitchhiking normalized to bare PLGA DiD NPs (from FACS, n=3, standard deviation), as % RBC

singlet population positive with at least one or more DiD NP/RBC. While 2HA notably confers hitchhiking ability on its own, stabilization of the coating by the choline cation on the PLGA surface enhances the number of DiD NPs present per RBC, shifting the intensity of the fluorescent-positive population on the RL-1 x-axis. A paired two-tailed t-test was performed between two samples at a time. * = $p<0.05$, **= $p<0.01$, ***=$p<0.001$. When baseline subtracted from saline and normalized to bare PLGA DiD NPs, CA+2HA 1:2 PLGA NPs show reduced RBC hitchhiking compared to CA2HA 1:2-PLGA NPs ($p=0.0004$). Comparatively, choline bicarbonate-coated PLGA NPs, assembled by electrostatics, demonstrate negatable RBC hitchhiking ($p=0.00016$), while 2HA-coated PLGA NPs, assembled by hydrogen bonding and disruption of the water solvation layer at the carboxylic-acid terminated PLGA interface,[54] demonstrate significantly higher RBC hitchhiking than CA+2HA 1:2 PLGA NPs ($p=0.0018$), albeit lower (with fewer NP events per RBC) than when combined with choline in IL form ($p=0.01$).

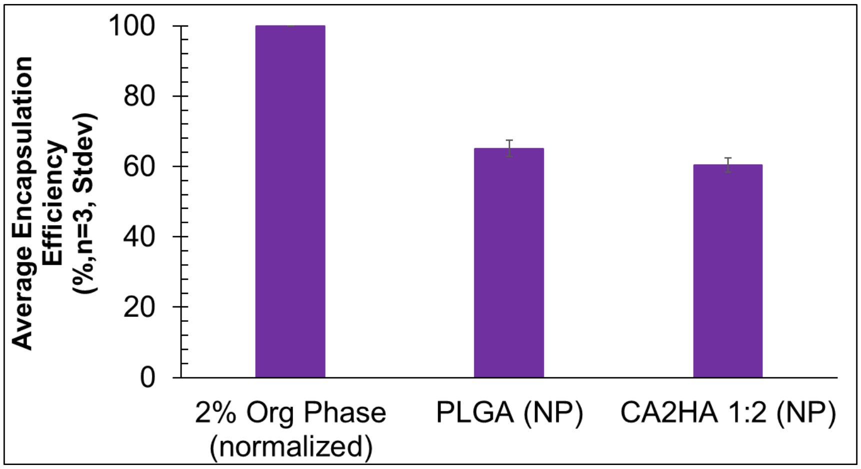

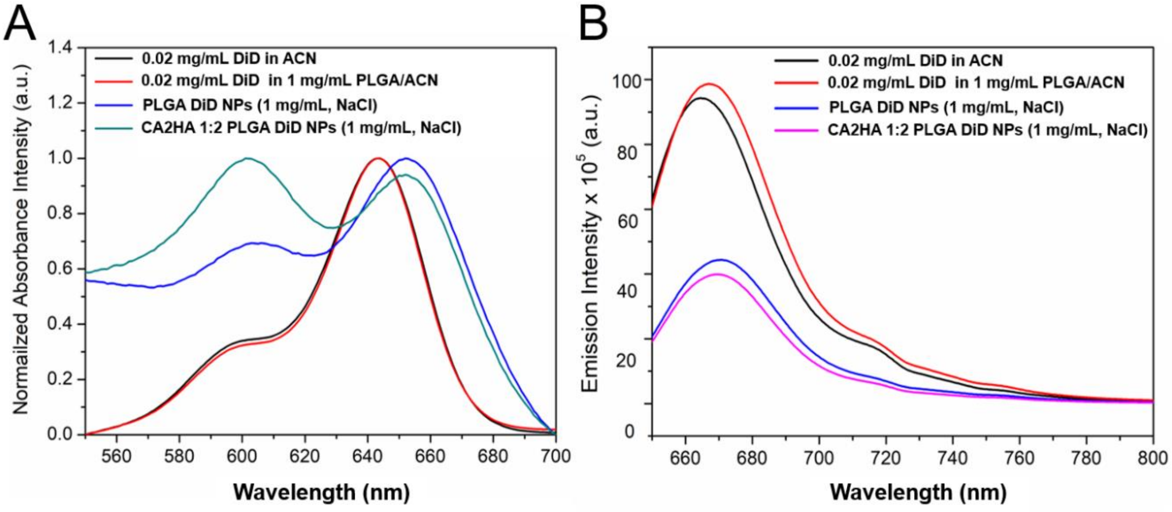

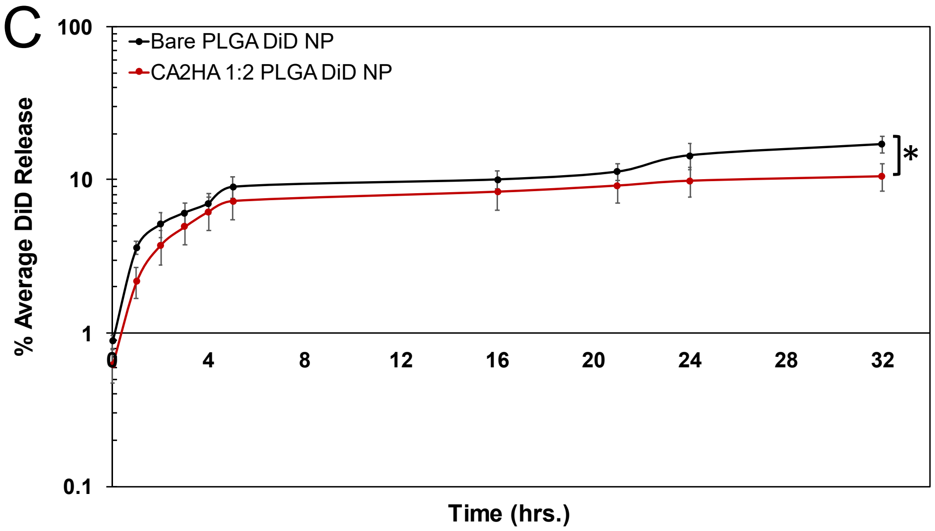

Figure S3. Encapsulation efficiency (EE), UV-Visible absorption spectra, fluorescent emission spectra, and 32-hour fluorescent release profile of DiD dye verify far-red DiD encapsulation in and comparable fluorescence between bare PLGA and IL-coated NPs with minimal free dye release. Fluorescence read across 200 µL NPs or Org.Phase/well). When normalized to the DiD organic phase used for synthesis (100%), CA2HA 1:2-coated PLGA DiD NPs yield an EE of 60.43 ± 2.03% (n=3). Bare PLGA DiD NPs yield an EE of 65.09 ± 2.29% (n=3).[46] (A) By UV-Vis., the two-phase absorbance (shoulder peak at 600 nm & characteristic peak at 640 nm) of DiD dye (red & black) slightly shifts as a result of molecular packing inside of both 1 mg/mL bare (blue) and IL-coated PLGA NPs (green) in USP-grade saline. The shoulder peak at 600 nm can be attributed to additional electronic transitions, vibrational modes, or formation of aggregates/excimers. Interestingly, CA2HA 1:2 further enhances the absorbance at 600 nm, compared to bare PLGA NPs, suggesting an additional compositional phase shift on the PLGA surface (where covalent dye interactions persist) upon IL coating. (B) The apex of the DiD fluorescent emission spectra (670 nm) is preserved from the 2% (wt/vol) organic phase (red) to the encapsulated bare (blue) and IL- coated PLGA NPs (pink), with an emission intensity trend comparable to EE via fluorescent plate reader. Interestingly, there is a noticeable red-shift observed when DiD is present together with PLGA in ACN solvent suggestive of the expected covalent binding between the dye and polymer, creating a new conformation of packed dye molecules around the polymer which is then encapsulated during nanoprecipitation (C) 32-hour fluorescent release profile of DiD from 1 mg/mL CA2HA 1:2-coated (n=4) or bare PLGA DiD NPs (n=3), in 0.9% saline, illustrates <10% total free DiD release for 5 hours, as well as a similar release profile up to 24 hours, with the IL coating conferring slightly more sustained release at 32 hours. *=$p<0.05$, **= $p<0.01$, ***=$p<0.001$.

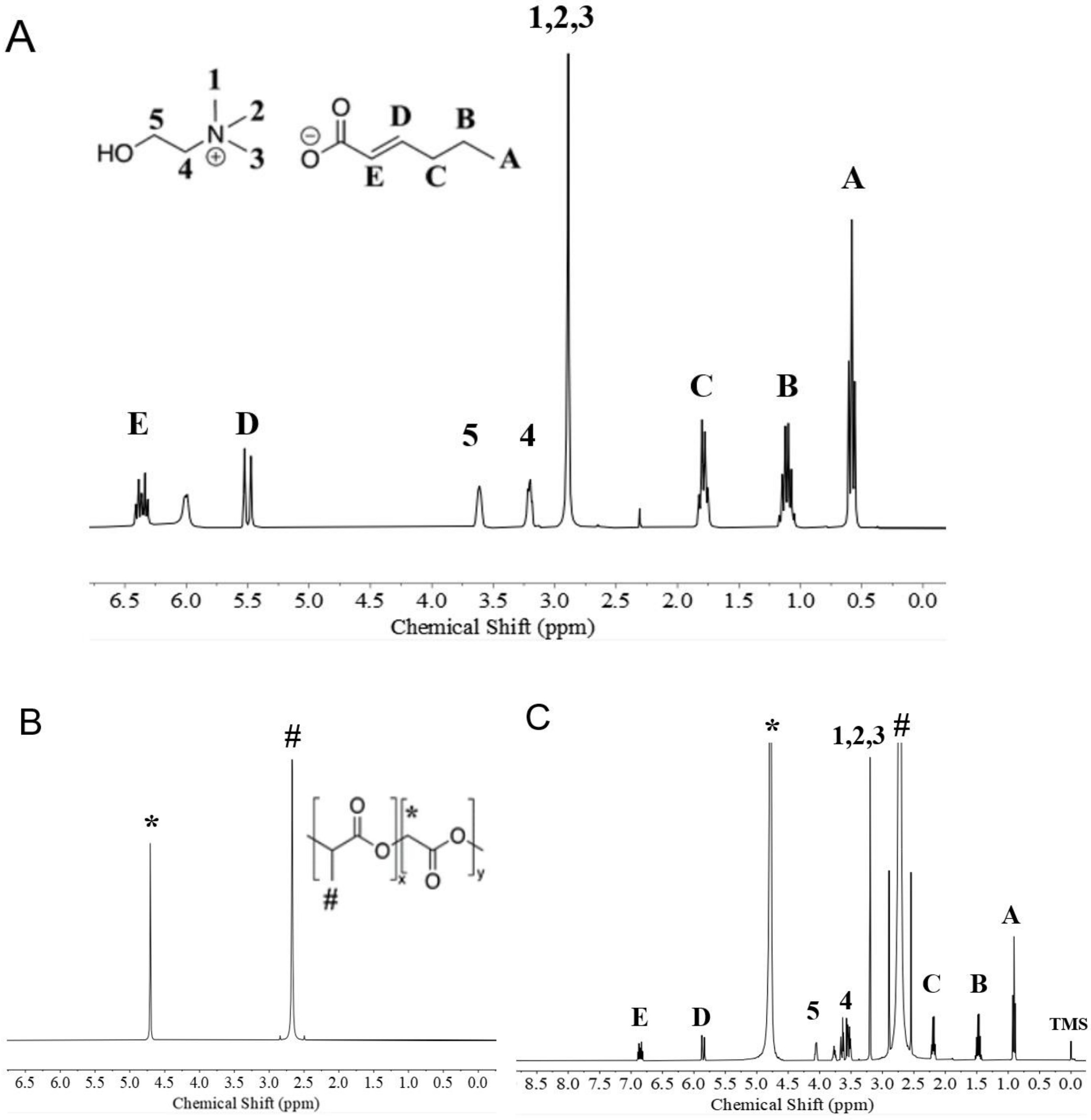

A
1,2,3
1
5
2
HO
N
3
4
D
B
E
C
A
E
D
5
4
C
B
6.5 6.0 5.5 5.0 4.5 4.0 3.5 3.0 2.5 2.0 1.5 1.0 0.5 0.0
Chemical Shift (ppm)
B
#
*
x
y
6.5 6.0 5.5 5.0 4.5 4.0 3.5 3.0 2.5 2.0 1.5 1.0 0.5 0.0
Chemical Shift (ppm)
C
*
1,2,3
#
A
C
B
E
D
5
4
TMS
8.5 8.0 7.5 7.0 6.5 6.0 5.5 5.0 4.5 4.0 3.5 3.0 2.5 2.0 1.5 1.0 0.5 0.0
Chemical Shift (ppm)

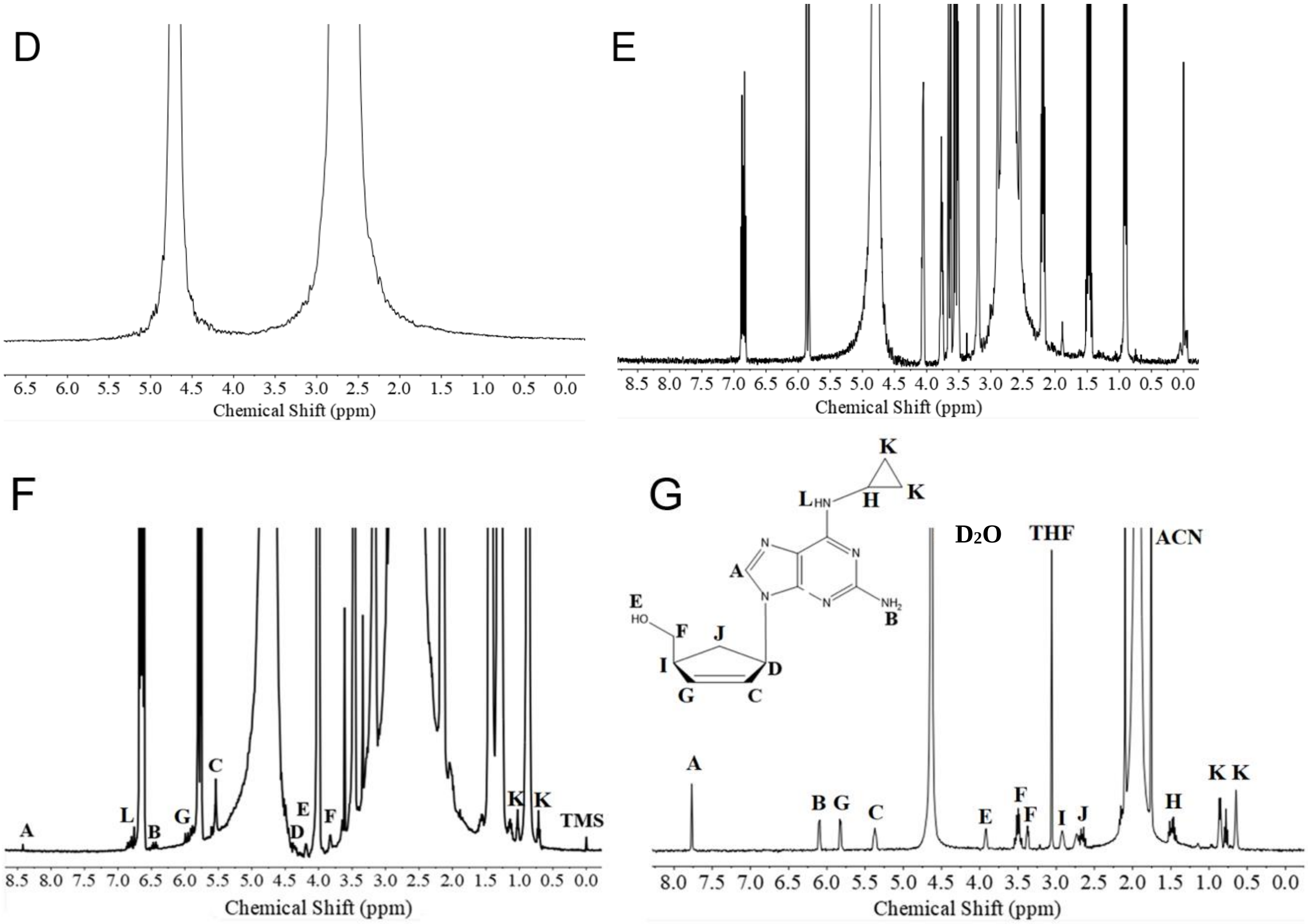

Figure S4. ^{1}H-NMR spectroscopy affirms CA2HA 1:2 IL coating on PLGA NPs and verifies presence of abacavir (ABC) ART when encapsulated inside IL-PLGA NPs. (A) ^{1}H NMR of choline and *trans*-2-hexenoic acid (CA2HA 1 : 2). (B) Intact PLGA NPs, (C) Intact IL-PLGA ABC NPs, zoomed-in baseline of intact (D) PLGA ABC and (E) IL-PLGA ABC NPs, and (F) destroyed IL-PLGA NPs revealing abacavir proton peaks, with 0.5 mg/mL abacavir drug reference in (G). (E) includes 20 μL TMS and (F) includes 2 μL TMS in D_2O (500 μL ~ 2 mg/mL) , respectively, for IL and ABC quantification.

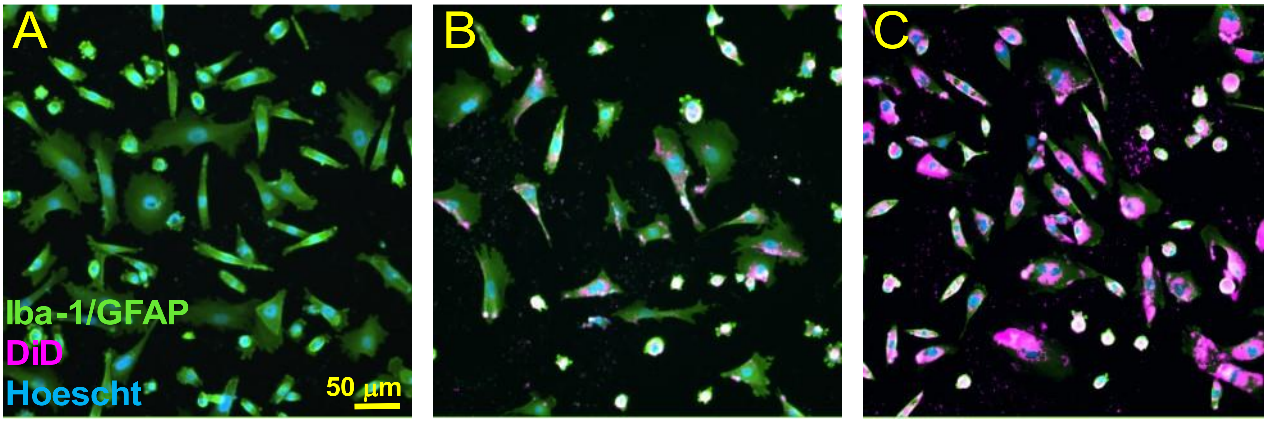

Figure S5. CA2HA 1:2 coating on PLGA DiD NPs enhances ex vivo uptake in co-cultured human microglia and human astrocytes. Immunocytochemistry on co-cultured human astrocyte and human microglial cells treated with (A) Media, (B) PLGA DiD NPs, and (C) IL-PLGA DiD NPs (purple). The cells were labeled with anti-Iba-1 or anti-GFAP antibodies (green) along with Hoechst nuclear counterstain. Intracellular DiD accumulation was qualitatively found to be greater when PLGA-NPs were coated with IL using a TI2-E motorized, inverted microscope (Nikon instrument Inc.).

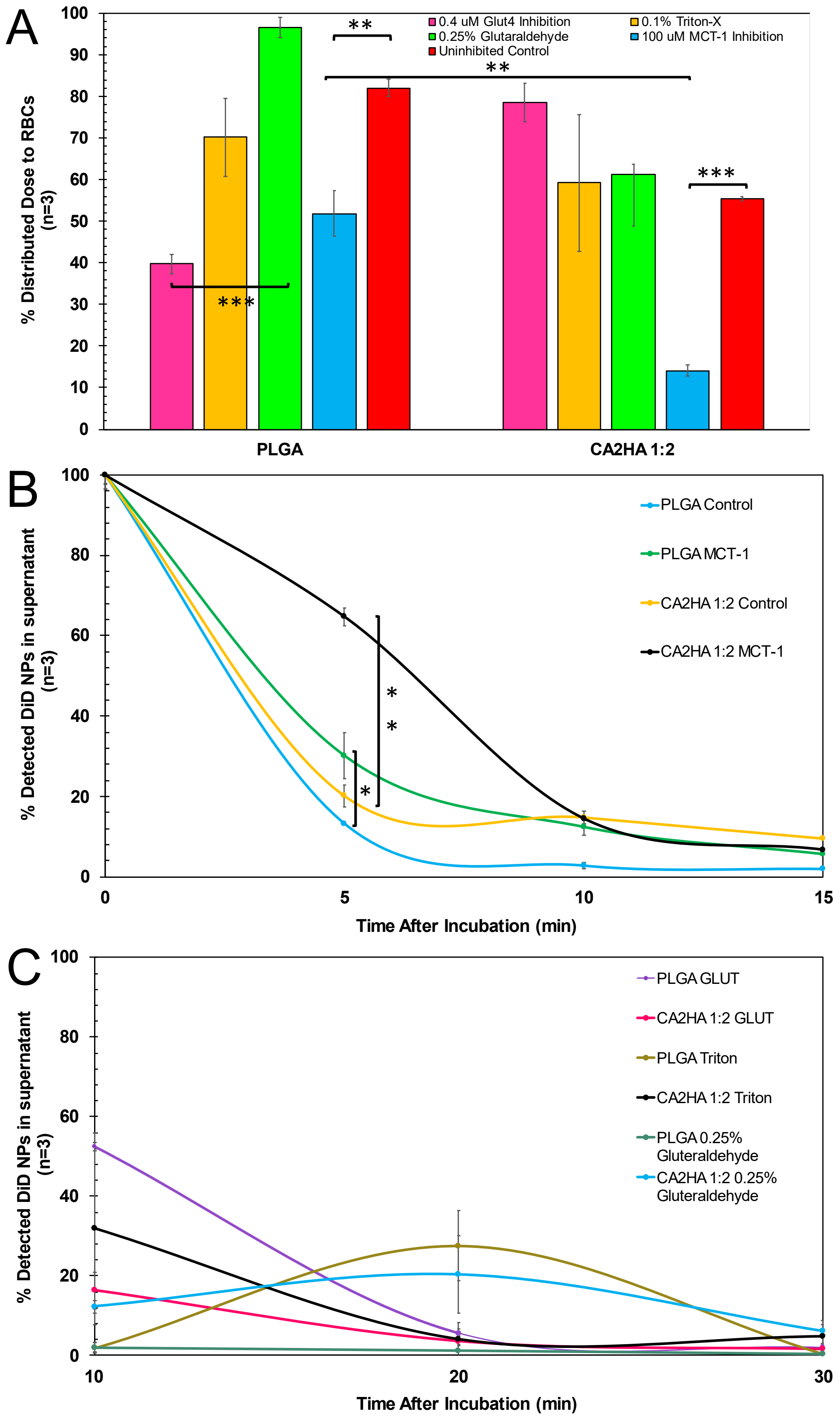
A
% Distributed Dose to RBCs (n=3)
0.4 uM Glut4 Inhibition
0.1% Triton-X
0.25% Glutaraldehyde
100 uM MCT-1 Inhibition
Uninhibited Control
0
10
20
30
40
50
60
70
80
90
100
***
**
**
***
PLGA
CA2HA 1:2
B
% Detected DiD NPs in supernatant (n=3)
PLGA Control
PLGA MCT-1
CA2HA 1:2 Control
CA2HA 1:2 MCT-1
0
20
40
60
80
100
**
*
0
5
10
15
Time After Incubation (min)
C
% Detected DiD NPs in supernatant (n=3)
PLGA GLUT
CA2HA 1:2 GLUT
PLGA Triton
CA2HA 1:2 Triton
PLGA 0.25% Gluteraldehyde
CA2HA 1:2 0.25% Gluteraldehyde
0
20
40
60
80
100
10
20
30
Time After Incubation (min)

Figure S6. MCT-1 may play a distinct role in driving CA2HA 1:2 chemical affinity to RBC membranes in whole murine blood, while PLGA affinity is more nonspecific and likely electrostatics and mechanically-driven. (A) % DiD NPs on washed RBCs after inhibition by 100 µM CHC (MCT-1), 0.4 µM Cyt.B (GLUT), 0.1% Triton, and 0.25% Glutaraldehyde. (B) DiD NPs that are detected in supernatant per wash after incubation with NPs after treatment with 100 µM CHC (MCT-1). (C) % DiD NPs that are detected in supernatant per wash after incubation with NPs after treatment with 0.4 µM Cyt.B (GLUT), 0.1% Triton, and 0.25% Glutaraldehyde. A paired two-tailed t-test was performed between two samples at a time, and ANOVA for 3 samples at a time. * = $p<0.05$, **= $p<0.01$, ***=$p<0.001$. While PLGA NPs are affected by MCT-1 inhibition due to their carboxylic acid surface termination ($p=0.0069$), they also have a non-specific inhibition by most treatment due to mechanical adhesion in the presence of isolated RBCs (ANOVA of uninhibited vs. GLUT, Triton, Glutaraldehyde; $p= 5 \times 10^{-6}$). Compared to its uninhibited control, CA2HA 1:2 is both more strongly inhibited ($p=0.0002$), and more selectively inhibited than PLGA ($p=0.0038$) by 100 µM CHC. Importantly, its binding is **not** affected by other aspects of the membrane dynamics (ANOVA of uninhibited vs. GLUT, Triton, Glutaraldehyde; $p= 0.11$).

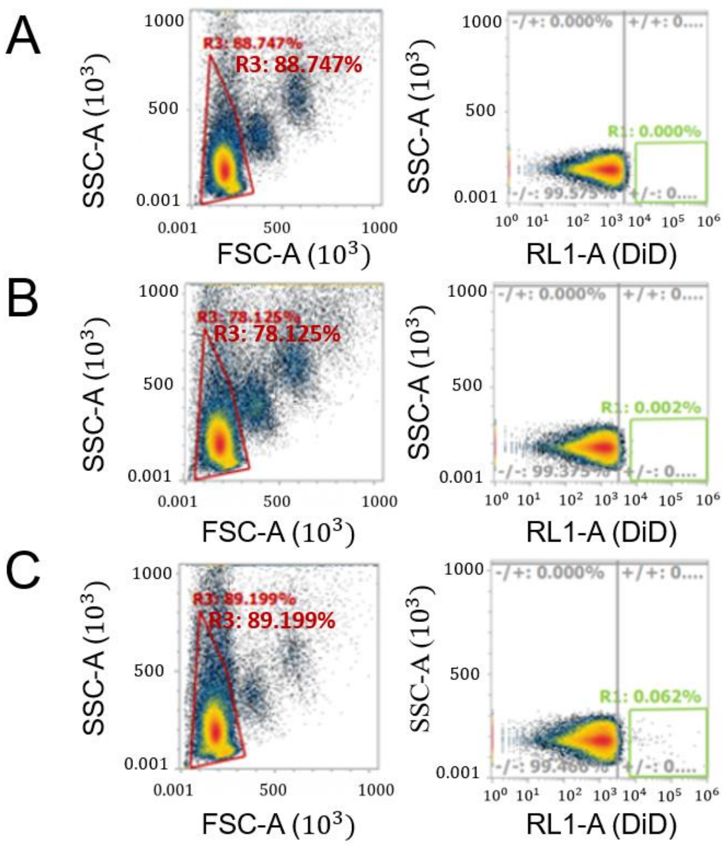

Figure S7. FACS detects only trace CA2HA 1:2 DiD PLGA NPs circulating on isolated RBCs from whole blood at the *in vivo* 6-hour timepoint, indicating NPs have sheared into blood-filtering organs and is secondary verification of first-pass accumulation into the brain. Pictured, left column: singlet-gated side vs. forward scattering profile of isolated RBCs from whole Sprague Dawley rat blood and right column: gated singlet RBCs vs. far-red fluorescence, from whole rat blood intravenously infused with (A) 0.9% saline (no fluorescence) , (B) bare PLGA DiD NPs (no fluorescence), and (C) CA2HA 1:2-coated PLGA DiD NPs (trace fluorescence).

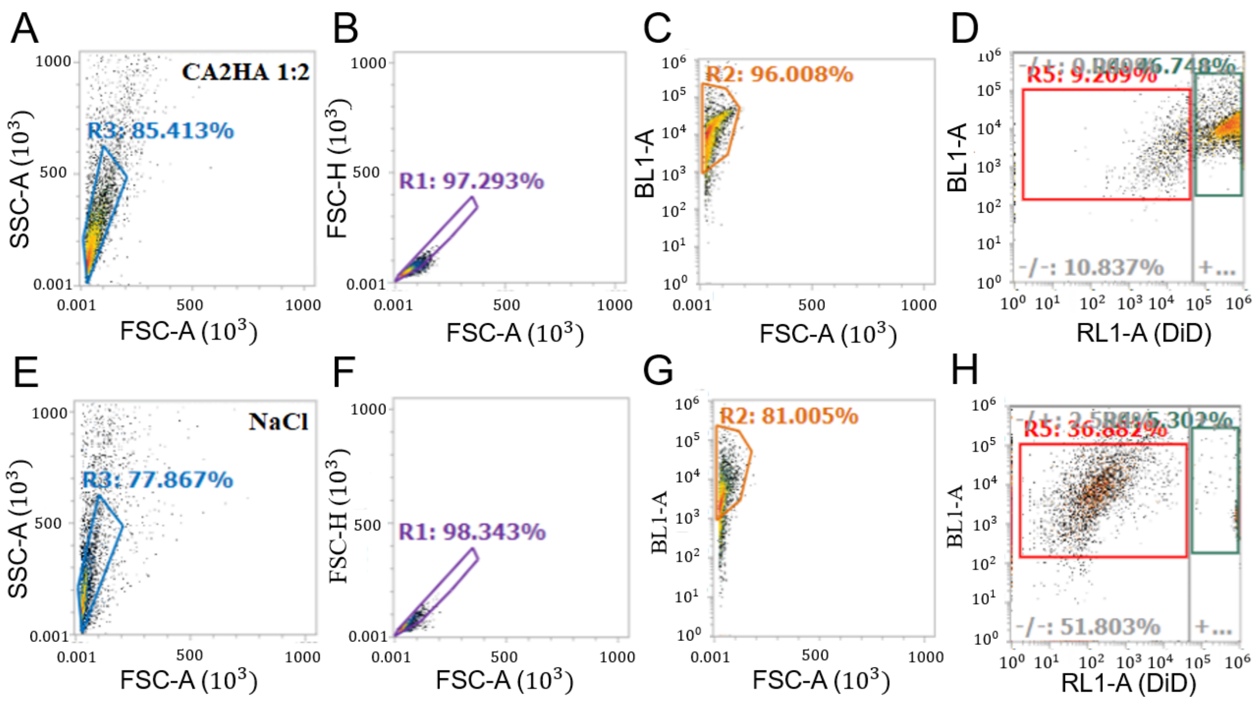

Figure S8. FACS of purified microglia isolated from CA2HA 1:2 DiD PLGA NP (top; A-D) and Saline (bottom; E-H)-treated Sprague Dawley whole rat brains verifies microglia-selective uptake at the *in vivo* 6-hour timepoint, verifying confocal microscopy of frozen brain sections. Pictured, left to right: side vs. forward scattering of isolated rat brain microglia, gated microglia singlet populations, FITC-CDllb+ positive forward scattering microglia, and FITC-CDllb+ microglia colocalized against far-red fluorescence (DiD).

References

[54] S. X. Edgecomb, C. M. Hamadani, A. Roberts, G. Taylor, A. Merrell, E. Suh, M. L. Yaddehige, I. Chandrasiri, D. L. Watkins, E. E. L. Tanner, Electrochem. Sci. Adv. 2023, e2300013.
